# Spatially resolved proteomics surveys the chemo‐refractory proteins related to high‐grade serous ovarian cancer

**DOI:** 10.1002/ctm2.70422

**Published:** 2025-07-23

**Authors:** Linyuan Fan, Yi Liu, Haichao Zhou, Yang Feng, Guangyi Jiang, Guixue Hou, Zhihan Cao, Zhiguo Zheng, Lu Sun, Hao Chen, Yuefei Zhang, Weiran Chen, Yun Xi, Benliang Cheng, Qinghai Yang, Yan Ren, Jianqing Zhu, Siqi Liu

**Affiliations:** ^1^ College of Life Sciences University of Chinese Academy of Sciences Beijing China; ^2^ BGI‐Shenzhen Shenzhen China; ^3^ HIM‐BGI Omics Center Hangzhou Institute of Medicine (HIM) Chinese Academy of Sciences Hangzhou China; ^4^ Zhejiang Cancer Hospital Hangzhou Institute of Medicine (HIM) Chinese Academy of Sciences Hangzhou China; ^5^ Fuzhou Maixin Biotech Inc. Fuzhou China; ^6^ Shanghai University of Traditional Chinese Medicine Shanghai China

**Keywords:** drug resistance, formalin‐fixed paraffin‐embedded, high‐grade serous ovarian carcinoma, laser capture microdissection, spatial proteomics

## Abstract

High‐grade serous ovarian carcinoma (HGSC) is a lethal malignancy characterized by high incidence, mortality, and chemoresistance. However, its molecular drivers are unknown. In this study, spatially resolved proteomics was applied to 1144 formalin‐fixed paraffin‐embedded tissue spots obtained by laser capture microdissection from 10 patients with HGSC and divergent carboplatin‐paclitaxel (CP) responses. Specific sampling revealed stroma‐driven tumour heterogeneity, identifying 642 tumour‐specific and 180 stroma‐specific proteins, with 505 CP‐responsive therapeutic targets. Most of these protein signatures represented previously unreported associations with chemoresistance in HGSCs. Two clinically significant spatial proteomic maps were generated by introducing tumour (TS) and chemical (CS) scores. TS analysis revealed conserved tissue architecture across CP response groups, whereas CS mapping revealed pretreatment metabolic reprogramming (rather than proliferation) as the defining feature of chemo‐resistant tumours, challenging current resistance paradigms. Immunohistochemical validation of HGSC tissue microarrays confirmed the spatial proteomic localization of TFRC and PDLIM3, which are linked to tumour progression, while establishing their novel role as chemotherapy resistance biomarkers through this study, with broader predictive potential observed across additional targets in the discovery cohort. This study developed a spatially resolved proteomic framework to enhance the diagnostic and therapeutic strategies for HGSC.

**Key points:**

- HGSC intra‐tumour heterogeneity is predominantly driven by stroma, as revealed by spatial proteomic compartmentalization (tumour/stroma).
- Spatial proteomics expands the therapeutic target database, enabling prediction of platinum‐based chemotherapy response.
- Chemo‐resistant patients exhibit pre‐treatment metabolic activation rather than proliferative signatures.
- TFRC (iron transport) and PDLIM3 (cytoskeletal remodelling) are spatially validated as chemo‐response biomarkers.

## Graphical abstract

1. HGSC intra‐tumour heterogeneity is predominantly driven by stroma, as revealed by spatial proteomic compartmentalization (tumour/stroma).
2. Spatial proteomics expands the therapeutic target database, enabling prediction of platinum‐based chemotherapy response.
3. Chemo‐resistant patients exhibit pre‐treatment metabolic activation rather than proliferative signatures.
4. TFRC (iron transport) and PDLIM3 (cytoskeletal remodelling) are spatially validated as chemo‐response biomarkers.

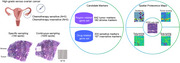

## 1 INTRODUCTION

High‐grade serous ovarian carcinoma (HGSC) is a prevalent malignant neoplasm in women with high rates of morbidity and mortality.^1^ Despite extensive research on the molecular pathology of HGSC,^2^ understanding of its molecular mechanisms from malignant tissues to the tumour microenvironment remains limited.^3^, ^4^, ^5^ Current knowledge is insufficient to address the challenges in clinical diagnosis and chemotherapy resistance in HGSC, which is a primary cause of treatment failure and recurrence in HGSC.^6^

The Cancer Genome Atlas (TCGA) has established solid evidence of large gene mutations found in tumour tissues, including HGSC, while improvements in RNA sequencing and protein identification by mass spectrometry have catalyzed the study of tumour‐related gene expression at the transcriptional or translational stage.^7^ The genomic alterations of HGSC have been thoroughly characterized and include ubiquitous TP53 mutations, massive copy number alterations (CNA), and mutations in homologous recombination genes such as BRCA1 and BRCA2.^8^, ^9^, ^10^ Transcriptomic responses to tumour development and chemotherapy in bulk HGSC tissues have been broadly investigated and found several dysregulated genes.^10^, ^11^, ^12^ Whilst traditional approaches fail to retain spatial information of gene expression at the subcellular level, single‐cell RNA sequencing (scRNA‐seq) and spatial transcriptomics have been deployed in HGSC research to address this.^13^, ^14^, ^15^ Zhang et al.^16^ reported that transcript profiles of 11 HGSC samples using scRNA‐seq revealed substantial cell clusters with transcriptomic variances in the patients who responded to chemotherapy. Denisenko et al.^17^ utilized the technology of spatial transcriptomics in 8 patients with HGSC and revealed the region‐dependent transcriptomes in stromal and immune cell populations. These findings provide a new perspective for understanding HGSC heterogeneity. However, whether the spatial characteristics appear at the protein level in the HGSC‐related tissues is unknown.

As proteins are mediators of biological functions, proteomics is instrumental in the HGSC research as well.^18^, ^19^ Zhang et al. conducted a multi‐omics analysis of 169 HGSC samples and emphasized that combined proteomics and genomics could provide insights into biological pathways and processes that drive ovarian cancer.^20^ Chowdhury et al.^21^ characterized the proteomic landscape of 242 HGSC samples and identified 64 protein signatures that could predict the HGSC resistance to initial platinum‐based therapy in two independent patient cohorts. However, traditional approaches in proteomics inherently face limitations when investigating protein interactions, spatial distributions, and spatial heterogeneity within tumour tissues, thereby limiting our understanding of protein characteristics related to HGSC. Compared with spatial transcriptomics, spatial proteomics technologies were developing faster. Hunt et al.^22^ utilized laser capture microdissection (LCM) and mass spectrometry to analyze the protein profiles of the tumour epithelium and tumour‐associated stroma in HGSC, revealing protein level heterogeneity in the tumour microenvironment. Eckert et al.^23^ employed a similar strategy to quantitatively analyze approximately 5000 proteins and to conduct differential proteomics analyses of the tumour and stroma regions in metastatic HGSC. Scalise et al.^24^ adopted a target approach to compare differences in the selected protein features in 23 platinum‐sensitive and 22 platinum‐resistant chemotherapy patients with HGSC and observed a close correlation between neutrophil infiltration into the tumour microenvironment (TME) and chemotherapy responses. Spatial proteomics is a promising approach in cancer research; however, its application in HGSC remains limited due to profiling constraints, the need for a large sample size and a lack of a comprehensive spatial view.

There are several technological challenges in the field of spatial proteomics for cancer. The first is the selection of appropriate materials. In clinical oncology, formalin‐fixed paraffin‐embedded (FFPE) samples are the most prevalent and extensively studied underpinnings for prospective and retrospective investigations. These materials maintain the integrity of molecular compositions and the original spatial architecture of tissues. Although freshly frozen specimens are commonly employed in spatial transcriptomics, the low efficiency of mRNA extraction from FFPE specimens impedes relevant results.^25^ However, noteworthy progress has been made in obtaining high‐quality proteomic output from FFPE samples.^26^ Second, the identification and quantification of proteomes in the minimized spots on FFPE samples in spatial proteomics is required, aiming at a special collection of microsamples with biological or pathological significance and global visualization of proteomic distribution. Instrument improvements in LCM and mass spectrometry have greatly accelerated their relevance in cancer research. For instance, Hu et al.^27^ developed the PLATO framework, which integrates microfluidics and deep learning to achieve high‐resolution proteome mapping across entire tissue sections. And our laboratory developed a workflow that enabled LCM sampling of the micro‐FFPE samples as small as.002 mm^2^ and identified over 2000 proteins on such small surfaces.^28^ Third, the generation of a spatial map for the proteome means a large sample from a micro‐surface and informatics analysis conducted for extensive proteomic data on the same sample. Conversely to the traditional approach, bioinformatics of the spatial proteome is expected to have specific characteristics, such as acquiring data from different spaces on the same sample, leading to relatively larger individual differences, and the construction of proteomic maps with parameters ignored by regular proteomics. Fortunately, the algorithms and evidence from single‐cell and spatial transcriptomes provide the potential solutions to these issues. Hence, in‐depth proteomic analyses of the surface of HGSC tissues promise a high resolution of spatial proteomes and greater comprehension of HGSC heterogeneity.

To construct a spatial proteome map of HGSC on FFPE tissues, an experimental strategy was carefully designed, involving the collection of the HGSC FFPE tissues with strong/weak chemotherapy responses under the strict judgment of a pathologist, excision of the micro‐FFPE tissues with LCM at tumour‐ or stroma‐specific regions and global surface, determination of proteomes in each excised micro‐FFPE spot, and analysis of space‐dependent or drug‐sensitive proteomes with multiple informatics tools. As a result, the first map of the spatial proteome of HGSC FFPE tissues was generated, demonstrating relatively consistent proteomes in the tumour and diverse protein compositions in the stroma regions of HGSC. Furthermore, comparing the spatial proteome maps of chemotherapy‐sensitive and chemotherapy‐insensitive HGSC tissues offers a new approach to identify candidates for chemotherapy‐sensitive proteins.

## 2 RESULTS

### 2.1 Overview towards sample collection and proteomic analysis on the HGSC FFPE tissues

**FIGURE 1:**
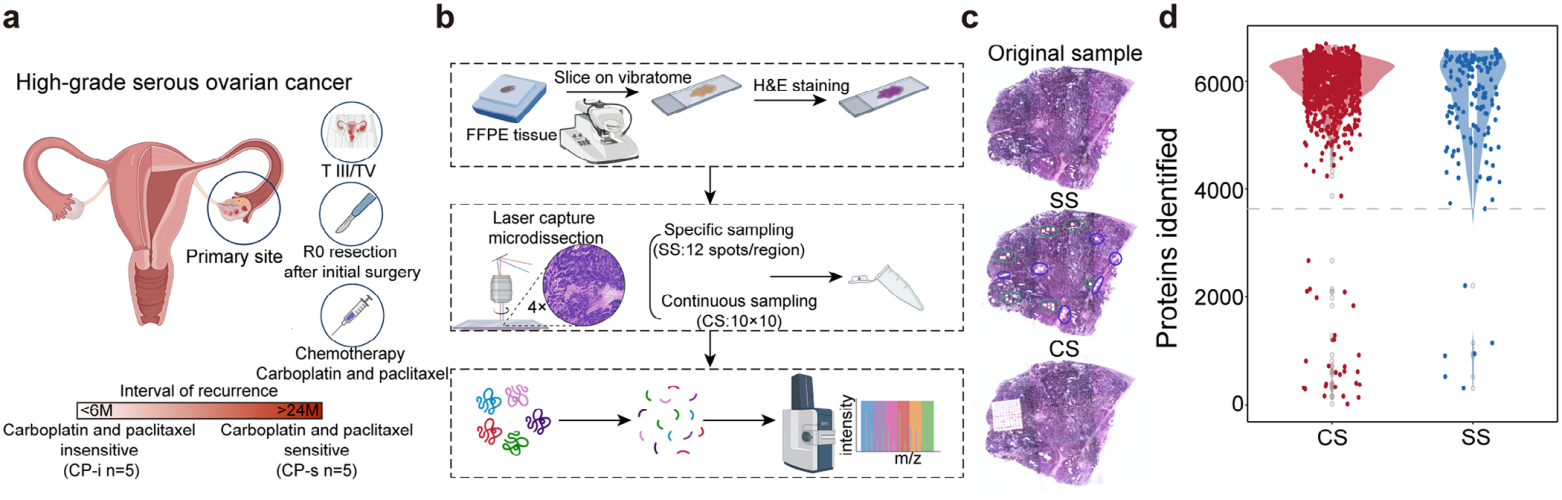
Schematic overview for spatial proteomics of HGSC. (A) HGSC patient collection. (B) Workflow of spatial proteomics analysis, including LCM sampling, decrosslinking, and tryptic digestion of micro‐FFPE tissues and (C) Image illustration for LCM sampling on micro‐FFPE tissues, specific sampling (SS) (Spots from tumor region marked with green circle, and from stroma region marked with blue circle with pathologist‐confirmed) and continuous sampling (CS), and (D) Distribution of proteins identified in all the spots in CS or SS groups (*n* = 1144 spots) for removal of the outlier spots with low identification rate (*n* = 40 spots).

To collect the clinical samples that truly reflect the response of HGSC patients to chemotherapy, the FFPE samples and clinical records of patients with HGSC in the Zhejiang Cancer Hospital were primarily screened following a schematic filtration, as illustrated in Figure 1A. Basically, an enrolled patient of HGSC should be matched with the criteria, (1) in the advanced stage, (2) at R0 level after the initial surgery, (3) six cycles of carboplatin and paclitaxel (CP) chemotherapy postoperative resection, and (4) clear pathological diagnosis on H&E staining images of surgical tissues. According to the platinum‐free interval (PFI), the patients were divided into CP‐s and CP‐i, in which the patient PFI values in CP‐s were more than 24 months after chemotherapy, while those in CP‐i were less than 6 months after chemotherapy. All the CP‐i patients died within 24 months, whereas all the CP‐s patients were alive before our last follow‐up at least 24 months after chemotherapy (Table 1; Supporting Information Data S1).

The workflow of spatial proteomics on FFPE tissues basically followed the method^28^ developed in our laboratory with some modifications as presented in Figure 1B, such as microdissection machines and spot transfer. Briefly, a micro‐FFPE sample with H&E staining was collected by laser capture microdissection (LCM) with 330 µm × 330 µm × 5 µm and the LCM‐based sampling on FFPE slide from a patient with HGSC was implemented in two modes, specifically picking up the regions recognized by pathologists as tumour or stroma (12 spots/region/sample) and continuously picking up spots on a relatively larger regions mixed with tumour and stroma (100 spots/slide, Figure S1A). The diagrams to indicate the HE images from the enrolled patient mentioned above and the spot excision mode are given in Figure 1C (all the details of HE images for the FFPE slides of HGSC patients in Supporting Information Images). The micro‐FFPE samples were treated with de‐crosslinked and digested. The digested peptides were delivered to a high‐resolution mass spectrometer to acquire MS/MS signals in the DIA mode. Proteomic data acquired from the micro‐FFPE samples were globally evaluated in terms of MS signal quality and quantitative normalized quantiles.

A total of 1144 spots from the FFPE tissue slides of ten patients with HGSC were picked for proteomic analysis using either specific or continuous sampling approaches. With the proteome of *E. coli* lysate as a control, the correlation of protein abundance of this control during such proteomic analysis on a large scale remained at.94, indicating acceptable data quality for protein identification and quantification generated from the MS platform (Figure S1B). In total, 8655 proteins were identified in these spots. The identified proteins in all the spots appeared in a typical Gaussian distribution, with an average of 5950 proteins identified per spot (Figure S1 C). Some spots with identified proteins at the outlier level were removed from the next step of analysis; thus, a total of 1104 spots were qualified for further bioinformatic analysis (Figure 1D). In those spots collected from the specific regions, approximately 11 spots per region were qualified as either tumour or stroma, while in those spots continuously sampled on the FFPE slides, over 97% of the spots per slide qualified for proteomic informatics through the data quality control. The proteomic data acquired from the micro‐FFPE samples were used to mine the spatial information of the proteomes in HGSC tissues.

### 2.2 Region‐specific features derived from proteomic analysis on HGSC FFPE tissues

**FIGURE 2:**
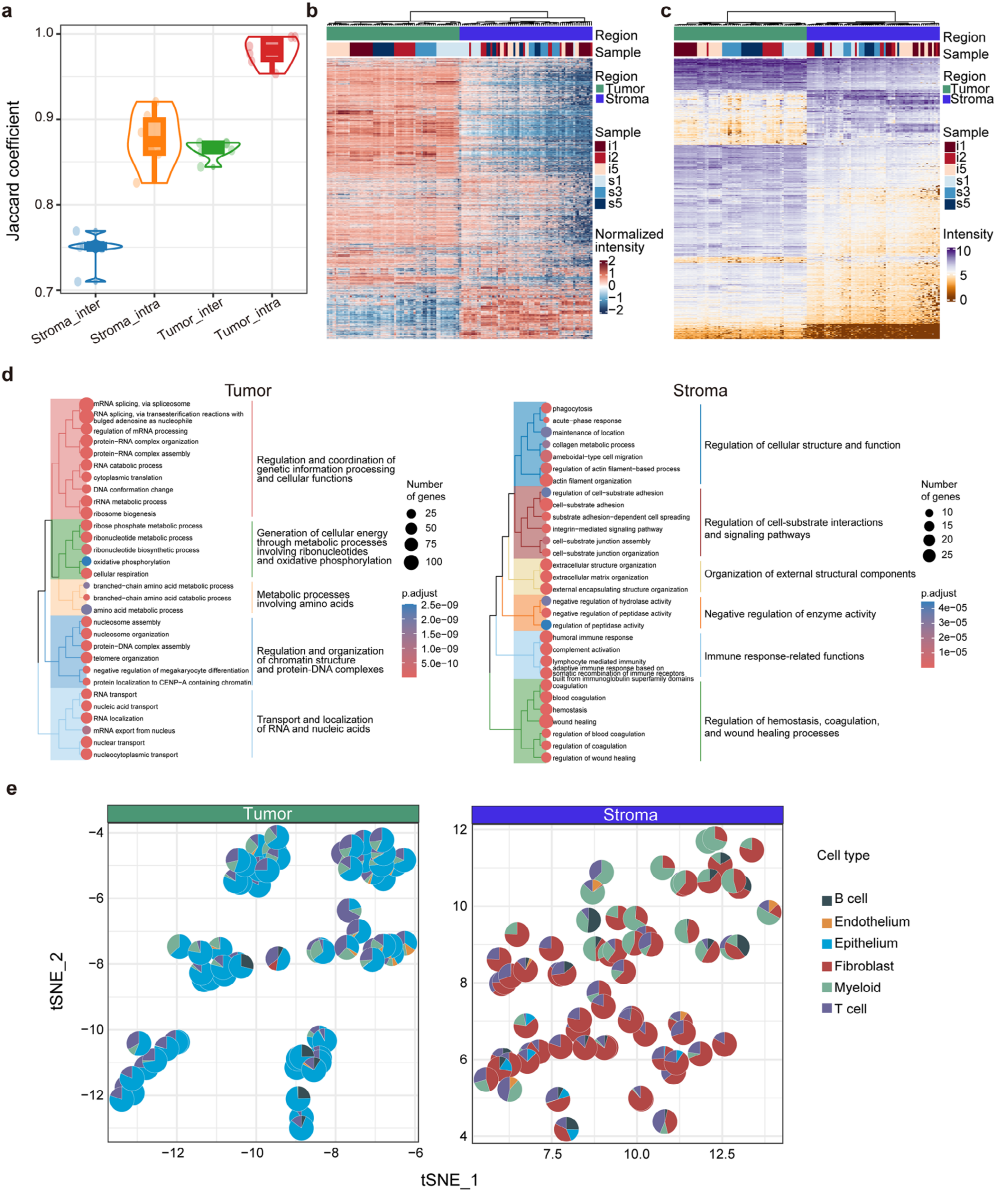
Qualitative and quantitative analysis towards the identified proteins in tumor and stroma regions of HGSC. (A) Evaluation of similarity or difference of the identified proteins among intra‐ or inter‐samples using Jaccard similarity. (B) Heatmap for all the identified and quantified proteins in tumor and stroma regions or (C) for all the DEPs between tumor and stroma in all the qualified spots. (D) GO‐BP analysis of all the upregulated DEPs in tumor or stroma, respectively. (E) Cell deconvolution for every spot either tumor or stroma in all the samples with spatial proteomics. A pie chart stands for the ratios of cell types labeled by different colors in a spot.

On average, the identified proteins per spot in the tumour regions were obviously higher than those in the stroma region (6270 vs. 5186). The overlap of identified proteins among spots in the tumour region of a sample ranged from.95 to.99, while the overlap between tumour regions in different samples reached.84 to.87. Conversely, the overlap rate for proteins identified among stroma spots within the same and across different samples was.83 to.92 and.71 to.77, respectively (Figure 2A). Moreover, the protein overlap status in the tumour and stroma was consistent among the CP‐s and CP‐i samples. The degree of overlap for the identified proteins was greater in tumour regions than in the stroma, suggesting higher protein consistency in tumours and indicating greater cellular complexity in the stroma. A closer examination of the unique proteins that were consistently detected in >50% of spots revealed 13 proteins in the tumour region and one in the stroma (Figure S2A,B). Of the 13 tumour‐unique proteins, ten were documented as tumour‐associated and two as HGSC‐associated, marking them as tumour features of HGSC. The stroma‐unique protein, CPZ, is noted as an enhanced biomarker in ovarian stromal cells based on the protein atlas and is associated with metallocarboxypeptidase activity in the extracellular space^29^ (Supporting Information Data S2). Thus, these unique proteins from either the tumour or stroma are considered candidates for protein biomarkers in specific regions of HGSC.

The quantitative information obtained from the region‐specific proteomes of HGSC was further analyzed informatically. As shown in Figure 2B and Figure S2D, all the spots collected from the tumour and stroma regions, including 3651 proteins with higher standard deviation and higher expression as shown in Figure S2C, were well divided into two groups using either principal component analysis (PCA) or K‐means clustering in the unsupervised mode, suggesting distinct proteomic characteristics between tumours and stroma. If clustering was focused on specific regions, tumour spots were clustered by sample, whereas the spots from the stroma were more dispersed than those from the tumour in Figure S3D. This suggests consistent proteomic features in tumours and variability in the stroma, reflecting a complex tumour microenvironment. Regardless of the tumour or stroma regions, it was necessary to remove individual differences caused by protein abundances in samples to identify differentially expressed proteins (DEP) between the two regions. For this purpose, random sampling was used to evaluate the protein abundance within individuals from all tumour or stroma spots, filtering proteins with significant differences in abundance. The pooled proteins, unaffected by individual sample variations, were subjected to DEP analysis, establishing a threshold of fold change ≥2 and adjusted *p*‐value < .05, obtaining 629 and 179 upregulated DEPs in the tumour and stroma, respectively (Supporting Information Data S2). A total of 822 DEPs (Figure 2C), including unique proteins (13 in the tumour and one in the stroma), were enriched based on GO‐BP (Figure 2D). Over 90% of the enriched pathways for tumour DEPs were associated with RNA processes, including RNA nucleotide synthesis, cell division, RNA splicing, and RNA transport, whereas 63% of the pathways for stroma DEPs were involved in cell‐cell interactions and immune responses. This was an expected result because in the tumour region, the higher proliferation of tumour cells is tightly associated with activated transcription processes in the S phase, and in the stroma region, complicated matrix cells and immune networks are well recognized. Moreover, a database of HGSC protein biomarkers was constructed considering published documents in the last 5 years,^18^, ^30^, ^31^, ^32^ which contains 2457 proteins. Overlap analysis in the tumour or stroma revealed 17% or.3% of the DEPs as published protein biomarkers, suggesting that potential HGSC protein biomarkers are likely to be derived from the tumour but not from the stroma, and the dataset generated in the study provides a valuable information source for mining HGSC protein biomarkers in future.

The next question was whether spatial proteomics could be used for cell deconvolution, and fine‐tune the cell distribution in different regions. As described in the Methods section, the deconvolution was divided in three steps: (1) getting 1746 proteins their abundance is highly correlated with transcripts in the region proteomics, and (2) obtaining 55 cell protein features based on scRNA database,^13^ and (3) cell deconvolution being conducted in CIBERSORT based on the cell protein features and protein abundance. With the information treatment, the cell distribution in the tumour or stroma regions was achieved (Figure 2E; Figure S2E). The results revealed that in the tumour region, the cells are mainly consisted of three types, with an average cell proportions per spots of 68% of epithelium, 20% of T cells and 9% myeloid, whereas in the stroma region, the cells were classified to five types, 60% of fibroblasts, 18% of myeloid, 16% of T cells, 4% of B cells, and 2% of epithelium. Furthermore, by checking the changes in cell proportion in the tumour region, the epithelium as the major cell type was consistently found in all spots, whereas, in the stroma, the cell ratios per spots, especially for fibroblasts, myeloid or T cells, are differed, suggesting a relatively stable composition of cells in the tumour and unstable cell composition in the stroma. This result agrees with those of the proteomics analysis described above. Therefore, the proteomics data and the corresponding cell analysis, therefore, support the fact with higher homogeneity in the HGSC tumour and larger heterogeneity in stroma.

### 2.3 Spatial proteome characteristics on HGSC FFPE tissues

**FIGURE 3:**
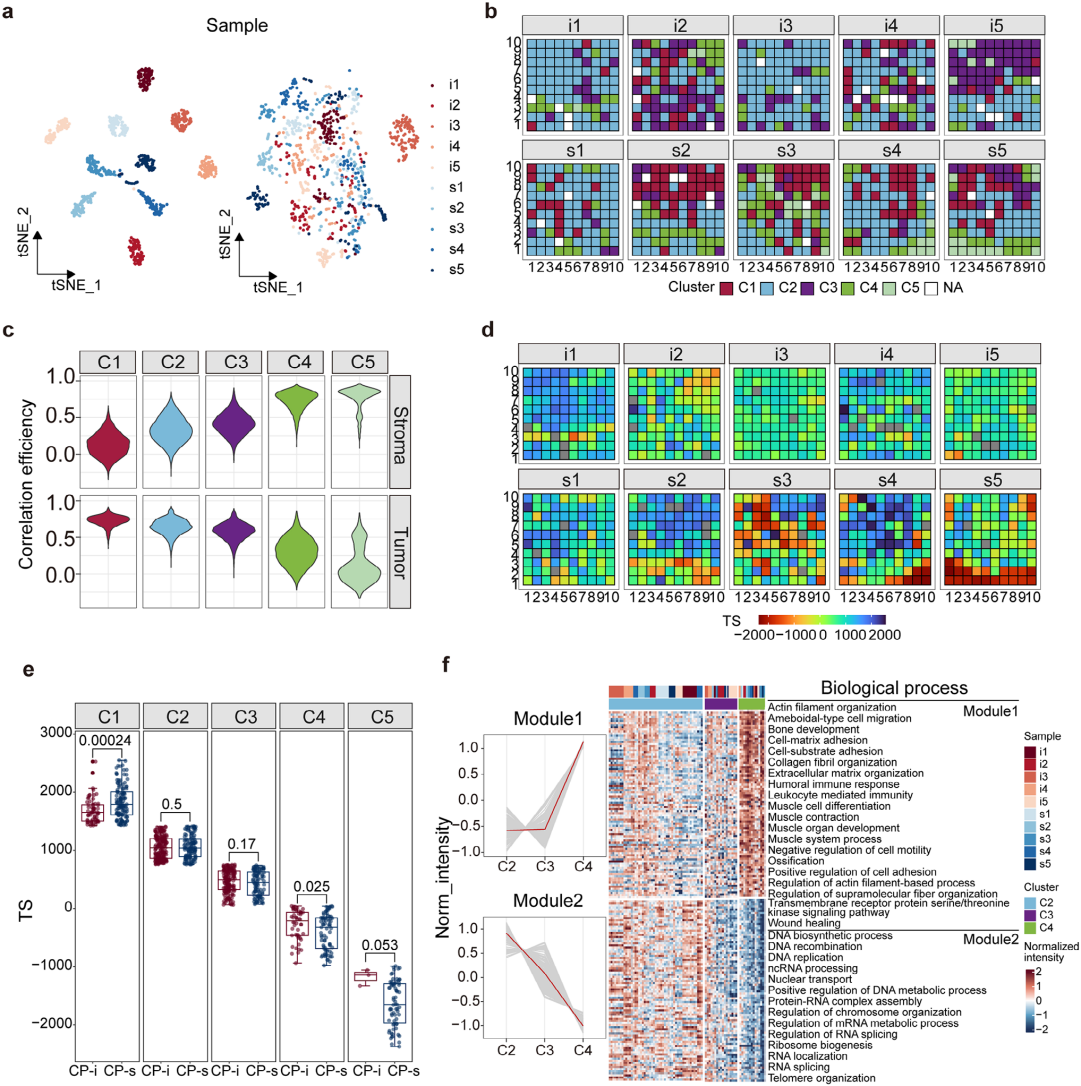
Generation of the spatial proteomics map of HGSC. (A) Comparison of the individual differences among samples by tSNE based on the proteomics data treated with (right) and without (left) Harmony treatment. (B) Spatial proteome map derived from Harmony treatments and unsupervised clustering. Spots labeled with NA were removed during the step of quality control. (C) Violin plot indicates abundance correlation for the proteins shared by the harmony clusters and tumor/stroma regions. The values of correlation efficiency close to 1 means high correlation and that close to 0 implies low correlation. (D) Spatial proteome map derived from TS. The bar of color gradient represents TS ranging from −2000 to 2000. (E) Analysis of significant difference between the paired clusters of CP‐i and CP‐s based on Wilcox test. And (F) GO‐BP analysis of the proteins in the cluster of C2, C3, and C4 that exhibit protein abundance with continuous increase or attenuation from C2 to C4.

Conversely to specific tumour or stroma regions, the continuously sampled spots in HGSC FFPE tissues contained mixed tumour and stroma cells. Spatial proteomics analysis involves assessing individual differences, defining the proteomic characteristics of each spot, and determining the spatial distribution of protein features. Proteomic data from these spots were processed using tSNE or K‐means clustering in an unsupervised mode. The tSNE results presented in Figure 3A show that the proteomic data generated from spots within the same sample were clearly clustered, indicating that interference in the proteomic data led to individual differences among the samples. To mitigate these problems, the harmony algorithm was applied to reduce the clustering degree (Figure 3A). PCA further refined the clustering into five categories (Figure S3A,B and 3B), with no significant differences in the cluster rates between the CP‐s and CP‐i groups (*p* < .01; Figure S3 C,D), indicating that the spatial proteomic characteristics were insensitive to chemotherapy. Random Forest analysis identified 450 proteins based on recursive feature elimination in these clusters (Figure S3E), revealing that the proteins in C1 correlated with tumour abundance, whereas those in C5 correlated with the stroma (Figure 3C). C1 and C5 occupied approximately 17% and 4% of the samples, indicating that C1 and C5 are termed tumour‐ and stroma‐like regions, respectively, with the tumour being more abundant than the stroma in HGSC FFPE samples.

Although the spatial proteome from HGSC FFPE samples was roughly clustered into five categories after the Harmony treatment, these categories did not directly reflect the distributions of tumour‐related proteins in the tissues. Single‐sample gene set enrichment analysis (ssGSEA) was used to characterize the positioned spots based on tumour‐related protein features, where the ssGSEA score indicated the similarity of DEP compositions in a spot related to those in the tumour or stroma. The tumour score (TS) is calculated by subtracting the ssGSEA score of stroma DEPs from that of tumour DEPs for each spot. As all the spots obtain their own TS values, the distribution of TSs is simply projected onto all the HGSC FFPE slides in this study (Figure 3D, S3F). The TS distribution for all the spots categorized into 5 clusters is depicted in Figure S3G, in which the highest peak value in the curves is found in C1 and the lowest peak value is perceived in C5, whereas, the peak values of C2 and C3 are close and that of C4 is very different from the other 4 clusters. The conclusion drawn from the TS is similar to that of Figure 3C, with C1 as the tumour‐like region and C5 as the stroma‐like region. Therefore, regardless of which approach is used to treat the spatial proteomics data, Harmony or TS, a similar profile of spatial proteomes is obtained in FFPE tissues. Because TS represents the overall DEP features in an excised spot, the question is whether the parameters could be used to judge the protein changes in response to drug sensitivity/insensitivity in tumour tissue. The Wilcoxon test was employed to compare TS scores in all the spots in the CP‐s and CP‐i groups with *p*‐value < .01. As shown in Figure 3E, except for C1, all the other clusters exhibited no significant differences between CP‐s and CP‐i groups. Notably, a significant difference is observed between C1 of CP‐s and CP‐i groups, implying that the DEP‐based parameters are sensitive to protein changes in the tumour region.

Considering that C1 and C5 are broadly grouped into tumour and stroma‐like regions, we sought to understand the molecular characteristics of C2, C3, and C4. To address this question, 450 proteins derived from Harmony were selected, and the corresponding abundance medium of these proteins was treated with fuzzy c‐means clustering for the spots of C2, C3, and C4. Then the proteins with trends of abundance changes, either consistently increasing or decreasing from these clusters, were chosen as the cluster indicators, with 294 proteins. Based on the GO‐BP for these cluster indicators, they were roughly annotated into two modes (Figure 3F) of consistent increase and decrease from C2 to C4, 149 and 145, respectively. A total of 149 proteins were enriched in the biological processes in nuclear location and functioning in DNA and RNA biosynthesis, while 145 proteins were annotated as functions related to extracellular matrix organization. The annotation of biological processes of these cluster‐associated proteins basically agrees with the trend of protein functions in C1 and C5. Because the TS values of C1 and C2 are similar, the two clusters share the functions in RNA processes, whereas C4 and C5 have similar TS values and likely perform the matrix functions in the stroma. The spatial proteome map shows the profiles of biological functions and pathways in HGSC FFPE tissues.

### 2.4 Assessment of CP‐resistant proteins on HGSC from the view of spatial proteomics

**FIGURE 4:**
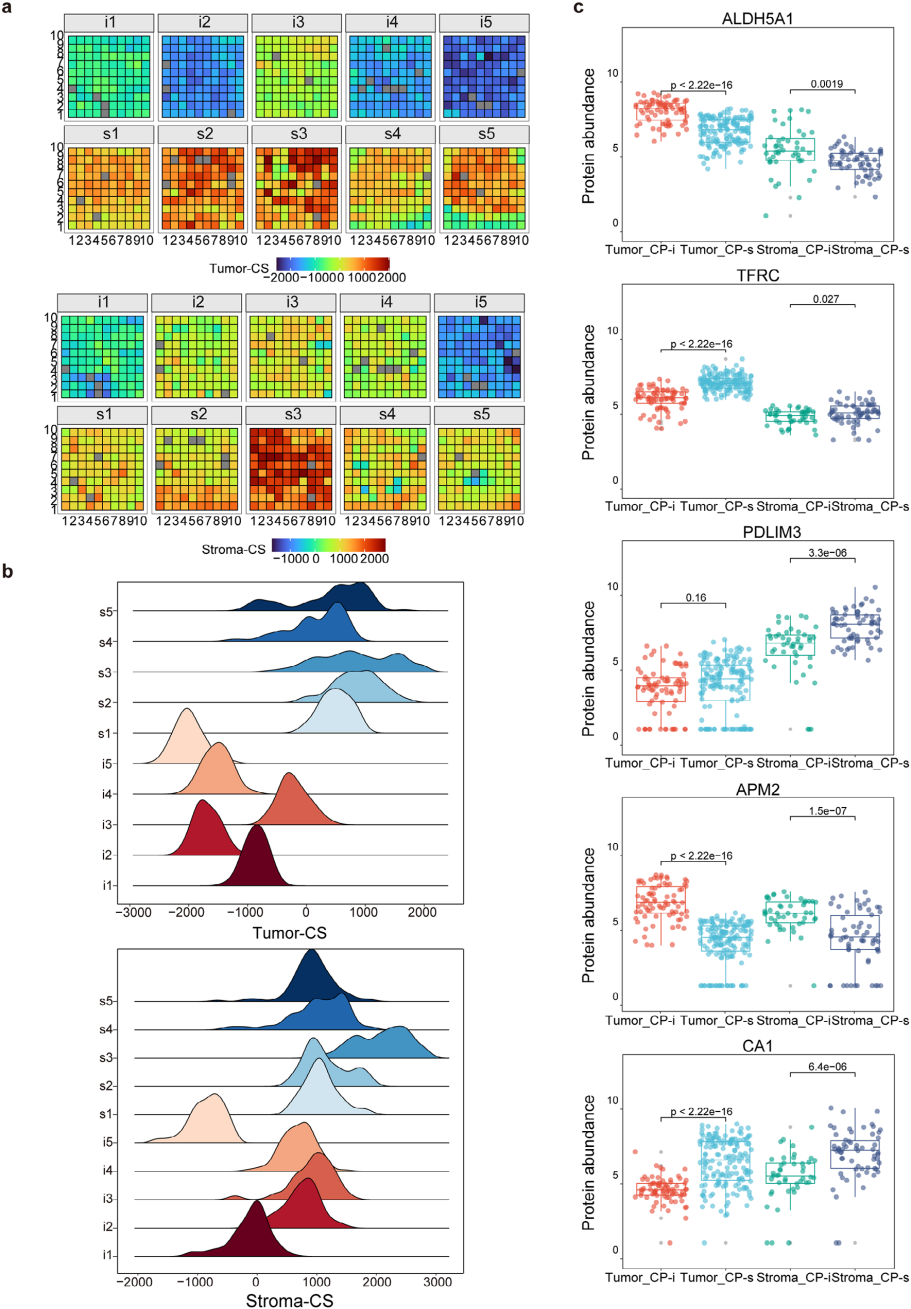
Exploration of CP‐sensitive or CP‐insensitive proteins on micro‐FFPE tissues in view of spatial proteomics. (A) Spatial proteome map derived from CS, upper for tumor proteins and lower for stroma proteins. The bar of color gradient represents tumor‐CS ranging from −2000 to 2000 and stroma‐CS scaling from −1000 to 2000. (B) Distribution of CS density in all the samples in the tumor or stroma. (C) Paired comparison for the protein abundance of five targets between CP‐i and CP‐s in all the samples in the tumor or stroma based on Student's *t*‐test.

As described above, the overall characteristics of spatial proteomes seem to lack obvious differences between the CP‐i and CP‐s of HGSC FFPE tissues, which prompts another angle to appreciate the protein changes in response to CP resistance in HGSC. Random sampling was performed to evaluate the differences in abundance between the spots of the CP‐i and CP‐s samples, removing those affected by individual variability. DEPs sensitive to CP (DEP‐cp) were defined based on fold change ≥2 and adjusted *p*‐value < .05. In the tumour regions, a total of 136 and 42 DEPs‐cp with upregulated abundance were marked in the CP‐i and CP‐s samples, respectively, whereas in the stroma regions, 61 and 266 DEPs‐cp were noted, respectively. Similar to the TS concept described above, ssGSEA was performed by determining the sets of proteins related to CP resistance, and the CP score (CS) was calculated by subtracting the ssGSEA score of the upregulated proteins in CP‐s from those in CP‐i. Each spot on the HGSC FFPE samples had its own CS; therefore, the CS distributions of either tumour‐CS or stroma‐CS were profiled in the CP‐i and CP‐s samples (Figure 4A). The distribution of tumour‐CS and stroma‐CS in each sample is shown in Figure 4B, indicating that the peak values of the CS distribution curve in CP‐i were globally lower than those in CP‐s. Following the clustering with Harmony and PCA, the significant differences in tumour‐CS or stroma‐CS in each cluster between CP‐i and CP‐s samples were evaluated by the Wilcoxon test, indicating that regardless of tumour‐CS or stroma‐CS, the values in all the clusters of CP‐s were greater than CP‐i (Figure S4A). Taken together, the TS distribution was very different from that of CS in CP‐i or CP‐s samples, and the generally higher CS values in the tumour and stroma in CP‐s samples imply some drug‐sensitive proteins being upregulated in these samples.

As the CS values derived from CP‐i samples were different from those of CP‐s, the question directly arises as to which proteins possibly affect drug sensitivity in tumour tissues. The C1 and C5 clusters were considered to be tumour‐ and stroma‐like (Figure 3C); thus, the DEPs‐cp in the two clusters could partially represent some functional responses to drug treatment in the tumour and stroma. GO‐BP analysis revealed that in the CP‐i samples, DEPs‐cp in C1 were enriched in energy‐related pathways, such as fatty acid and carbohydrate metabolism, whereas those in C5 were mainly involved in lipid metabolism (Figure S4B). In the CP‐s samples, the upregulated proteins in C1 focused on cell cycle processes, whereas those in C5 were related to matrix functions, such as the muscle system and hemostasis. The high proliferation of tumour cells is balanced by activated cell cycling activity and large energy requirements supported by enhanced metabolism. The pharmacological functions of CP are considered as the cell cycling inhibitor. Tumour cells with upregulated proteins involved in cell proliferation are thought to be targeted by CP, whereas those with a higher abundance of proteins involved in energy metabolism may not be specifically bound by CP. A review of the literature on CP in ovarian cancers revealed that only 34 of 660 CP‐related proteins were co‐identified in this study,^21^, ^33^, ^34^, ^35^, ^36^, ^37^ indicating a mere 3% overlap with a higher overlap among the publications, approximately 0–1.5%, possibly due to sample collection (fresh or FFPE), protein quantification (label or label‐free), or cohort size. For this study, spatial proteomics data were obtained by strict filtration to minimize individual differences in proteins, statistical quantification from multiple spots in the same region of different samples, and the DEPs‐cp with 86% of co‐identification and quantification in over half of the relevant regions and samples. The results of this study present a set of reliable quantification methods for exploring the CP‐related proteins in HGSC tissues.

A reasonable goal for the discovery of chemotherapy‐sensitive proteome is to find out several biomarkers with clinical value. Based on these CP‐related proteins, two experiments were considered to select potential CP indicators: the proteomic data to be further verified using different biochemical methods, and only a few candidates to be chosen in a clinical test of a large cohort. Prior to experimental confirmation, several targets were expected through stepwise filtration from the 491 DEPs‐cp. The filtration criteria for the verification targets were set up as (1) most of them are novel indicators without previous reports regarding their chemoresistance in HGSC, (2) as the region representatives of either tumour or stroma in this study, (3) higher abundance changes between CP‐i and CP‐s groups (fold‐change > 2, adjusted *p*‐value < .05), and (4) clear definition of biochemical functions in UniProt/Swiss‐Prot, such as TFRC^38^ and PDLIM3.^39^ Based on these criteria, five targets were finally chosen: TFRC, ALDH5A1, PDLIM3, CA1 and APM2. TFRC and ALDH5A1 were located in the tumour, PDLIM3 in the stroma, and CA1 and APM2 in both regions. The abundance differences of the five targets towards all spots in the tumour‐ and stroma‐like clusters between CP‐i and CP‐s were estimated using Student's *t*‐test. The statistical results are summarized in Figure 4C and Figure S4C, wherein ALDH5A1 and TFRC were sensitive to CP in the tumour, ALDH5A1 was upregulated in CP‐i and TFRC was upregulated in CP‐s. PDLIM3 was significantly upregulated in the stroma of CP‐s, but no change was observed between CP‐i and CP‐s in the tumour, and CA1 and APM2 remained in a similar mode of abundance changes in the tumour and stroma between CP‐i and CP‐s groups. From a global comparison of spatial proteomics to that of individual spots for the selected targets, all the data endorsed the five targets that were valuable for the next experimental verification.

### 2.5 Potentially CP‐sensitive indicators verified by tissue microarray with immunohistochemistry

**FIGURE 5:**
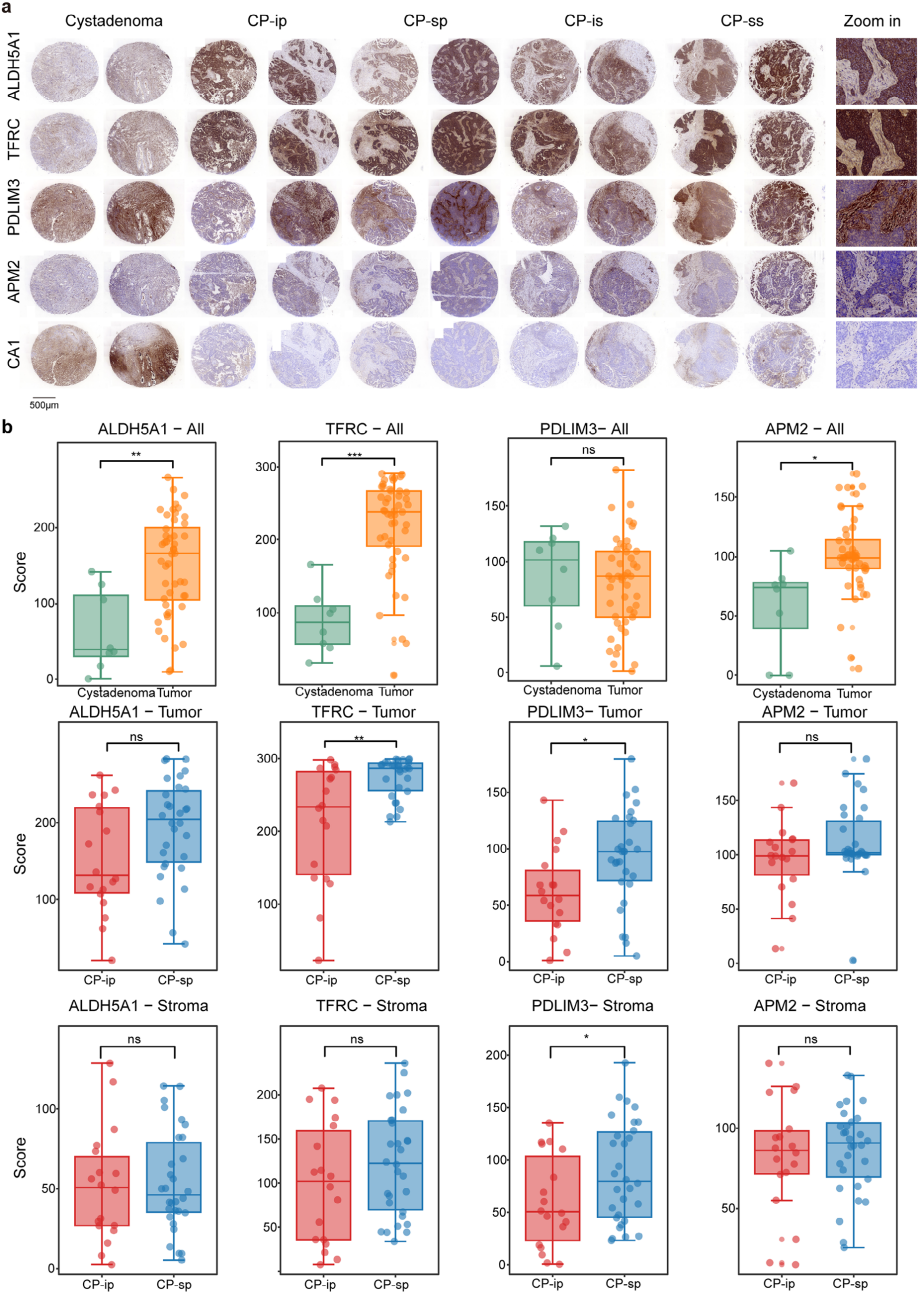
Typical IHC images and statistical estimation of the IHC staining signals on the tissue microarray. (A) The typical IHC images on the tissue microarray of HGSC. The samples on the tissue microarray are grouped into 5, CP‐ip (CP‐insensitive in primary HGSC), CP‐sp (CP‐sensitive in primary HGSC), CP‐is (CP‐insensitive in secondary HGSC), CP‐ss (CP‐sensitive in secondary HGSC) and cystadenoma. Two typical IHC images in each group are selected. On the right image panel, the localized IHC images with 5X amplification obtained from the sample in CP‐sp (on right). (B) Paired comparison for the IHC staining intensities of four targets between CP‐ip and CP‐sp in all the samples of the tissue microarrays based on Student's *t*‐test. On the upper panel, comparison of four targets between cystadenoma and tumor, and on middle and lower panel, comparison of four targets between CP‐ip and CP‐sp from tumor and stroma, respectively. The signs of *, **, ***, and ns represent a significance with *p* < .05, *p* < .01, *p* < .001, and non‐significance.

The tissue microarray was prepared in the laboratory of Zhejiang Cancer Hospital, which consisted of 91 samples, including five patient groups: 36 samples of primary CP‐s (CP‐sp), 18 primary CP‐i (CP‐ip), 13 secondary CP‐s (CP‐ss), 15 secondary CP‐i (CP‐is), and 9 benign masses (cystadenoma). Typical immunohistochemistry (IHC) images are shown in Figure 5A, wherein the samples (two from each group) were treated with five target antibodies. By assessing the performance of immunohistochemical staining, CA1 showed poor staining either in staining consistency in these samples or in weak recognition of tissue regions, whereas the other antibodies displayed good staining signals, indicating relatively stronger signals in tumour tissue than those in cystadenoma. Specifically, in the staining images of the tumour and stroma regions, ALDH5A1, TFRC, and PDLIM3 showed different staining intensities between the two regions, whereas APM2 exhibited poor recognition in differentiating tumour from stroma (Figure 5A). The quality of the IHC images was evaluated using HALO software.^40^ Of the image quantitation, the CA1 values were low with large diversity in the spots of both tumour and stroma, which were difficult to statistically assess and were not included in the next steps of data analysis; however, the other four targets were accepted by further quantitative statistics.

Based on the intensity of the stained area, significant differences between the paired samples were assessed using Student's t‐test (Figure 5B). Comparison of IHC signals between tumours and cystadenomas revealed that ALDH5A1, TFRC, and APM2 exhibited significantly more abundant HGSC‐related characteristics in tumours than in cystadenomas, whereas changes in PDLIM3 abundance were not found between tumours and cystadenomas. Further examination of the IHC signals in the tumour and stroma between CP‐i and CP‐s revealed that TFRC and PDLIM3 displayed significantly increased abundance in CP‐s, but not in CP‐i, and ALDH5A1 and APM showed no significant changes between CP‐i and CP‐s. In the stroma regions, only PDLIM3 displayed an abundant increase in the CP‐s, whereas the other three targets showed similar abundance patterns between CP‐i and CP‐s. Surprisingly, compared with spatial proteomics, the IHC signals of the four potentially CP‐sensitive targets are not in agreement with the abundance changes recorded by mass spectrometry. For TFRC, its IHC results are similar to its MS in the tumour, indicating that as a tumour biomarker, its abundance sensitively responds to CP treatment, while the changes of IHC and MS signals, in stroma, are not at the same pace, wherein no alteration in IHC and a slight increase in MS were detected. For PDLIM3, the results of IHC in the stroma are comparable to those of MS, and its abundant responses to CP in IHC in the tumour are different from its signals gained from MS PDLIM3 increases in CP‐s in IHC but no change in MS. For ALDH5A1 and APM2, although their MS signals were attenuated in CP‐s both tumour and stroma, their IHC signals were not significantly different between CP‐i and CP‐s, regardless of tumour or stroma. Evidence of spatial proteomics derived from MS is solidly endorsed by IHC data for general comparison of target abundance distribution and location in different regions; however, the assessment conclusions towards the target abundant responses to CP treatment in different regions in IHC partially disagree with those observed by MS. The disagreement between IHC and MS results is likely due to differences of measurement approaches and quantitative calculations. The Kaplan–Meier database contains HGSC samples along with their corresponding mRNAs and clinical information. Once the mRNA abundance of TFRC and PDLIM3 was downloaded from the database, it was applied to survival analyses on progression‐free survival (PFS) and overall survival (OS). The analysis indicates that higher TFRC mRNA abundance is associated with longer PFS and OS, while lower PDLIM3 mRNA abundance is responsive with longer PFS and OS (Figure S6). Obviously, the survival correlation with TFRC and PDLIM3 at the protein level is different from that of the two genes at the mRNA level. The conclusion conflict induced by protein image and mRNA array is difficult to attribute to certain factors, such as expression characteristics of protein and mRNA, sample size difference, sample source difference, and survival periods. However, the indicative roles of TFRC and PDLIM3 were strongly endorsed by solid data of spatial proteomics and IHC images. Therefore, on technological consideration, TFRC and PDLIM3 are acceptable for further extensive examination of CP‐sensitive HGSC tissues.

To evaluate the target performance in primary and secondary HGSC, a comparison of IHC images and target abundance was conducted in these samples (Figure 5A; Figure S5). In all the IHC images of the four targets, the corresponding antibodies recognized the tumour and/or stroma well; nevertheless, they did not appear to have significantly different IHC signals for discriminating CP‐i from CP‐s. There was no significant difference in the quantitative IHC signals between CP‐i and CP‐s. This suggests that the tumour or stroma regions in secondary HGSC possess different protein characteristics from those in primary HGSC, wherein the proteins involved in drug metabolism in the recurrent tissues are altered.

## 3 DISCUSSION

Profiling the spatial transcriptome or proteome of tissues can significantly enhance our understanding of tumour heterogeneity at the gene expression level. Although spatial transcriptomic atlases of HGSC have been established, spatially resolved proteomic profiling has remained absent in the literature, particularly for the studies linking subregional protein dynamics to clinical outcomes like chemoresistance. To fill in this gap, this study aimed to build a precise spatial proteome map of HGSC tissues, marking the first attempt to map the spatial distribution of HGSC‐related proteomes. Conversely to early studies on HGSC that used proteomic technology, our study provides unique contributions to the exploration of spatial characteristics and chemo‐refractory proteomes. First, by carefully recruiting FFPE samples from HGSC patients with varying responses to postoperative chemotherapy and employing an innovative LCM approach for both random and continuous sampling of the tumour and stroma regions, we generated a comprehensive spatial proteome map that aids in the global assessment of tumour and chemo‐refractory biomarkers. However, it is necessary to point out that for deep analysis of proteomics, approximately over 100 spots excised from a FFPE sample, a cohort study with large samples seems infeasible based on the current technology. Therefore, with the pathologist's help, the typical HGSC samples were collected to ensure their representativeness for such tumours, with 10 HGSC samples in this study. As a matter of fact, spatial proteomic evidence demonstrates relatively higher overlap of proteomic data among inter‐ or intra‐spots from tumour regions, implying a successful strategy in sample collection. On the other hand, it is a research direction in the near future that the conclusion deduced from the spatial proteomics should be validated in a large cohort of HGSC. Second, to address the challenge of minimizing individual proteome differences in data from multiple spots within the same sample, de‐batch methods were implemented, including random sampling statistics and harmony analysis, resulting in the generation of a spatially resolved proteome map for HGSC that highlighted DEPs between specific regions, tumour and stroma, or CP‐i and CP‐s. Third, to represent protein features on the HGSC FFPE tissue surface, particularly in continuously sampled regions, the ssGSEA method was employed to compute ssGSEA scores for each spot, enabling the characterization of tumour and CP resistance features, such as TS and CS, and facilitating the construction of spatially resolved maps of the HGSC proteomes. Fourth, to confirm the conclusions drawn from the spatial proteomics of HGSC, a tissue microarray with IHC was performed on five targets with significant abundance differences between CP‐i and CP‐s, reinforcing two targets as ideal candidates for CP sensitivity. The IHC findings generally supported the spatial proteomics analysis, despite some discrepancies.

Most HGSC surgical samples were extensively invaded by tumour cells, leaving little or no adjacent or normal tissue. Consequently, assessment relies heavily on histological analysis using hematoxylin and eosin (H&E) staining and microscopy, which allows for the identification of typical tumour or stroma regions and atypical transitional areas. In this study, the proteomic characteristics were first focused on the two specific regions, tumour and stroma, revealing a total of 642 tumour‐associated proteins, of which approximately 17% matched with the ovarian cancer biomarkers reported in previous studies, whereas less than 1% of DEPs in the stroma overlapped with previous investigations. This highlights the power of spatial proteomics in capturing the unique characteristics of tumours and explaining the complexity of the tumour microenvironment. In the analysis of drug resistance features using spatial proteomics, comparison of the spot proteomes in the tumour‐like and stroma‐like regions sampled in continuous mode between CP‐i and CP‐s resulted in 505 CP‐related DEPs with high reliability and stability in the experiment. However, the overlap rate between these DEPs and the drug‐related proteins in ovarian cancer was low. The reproducibility of the drug‐related proteins reported by different laboratories is very inconsistent as well, possibly because previous bulk‐proteomics studies did not explicitly clarify the heterogeneity of the clinical samples. The solid evidence from the spatial proteomics in this study contributes to expanding the dataset of the CP‐related proteins in HGSC. Importantly, the sensitivity of these proteins to chemotherapy was partially confirmed by an alternative approach using a tissue microarray with IHC. Therefore, spatial proteomics is a powerful tool for exploring the drug resistance targets.

Platinum‐based chemotherapy is commonly used for various cancers, including HGSC; however, platinum resistance is a significant challenge, with only a few related biomarkers identified.^41^, ^42^ The KEGG database mentions only one “Platinum drug resistance pathway,” primarily involving nucleotide excision repair, cell cycle, and apoptosis. In HGSC, spatial proteomics revealed that proteins associated with CP sensitivity in CP‐s tumours were primarily linked to the cell cycle, whereas resistant proteins in CP‐i tumours were enriched in metabolic pathways, indicating that CP effectiveness depends on tumour cells being in the proliferation stage of tumour. Furthermore, five targets associated with CP‐related features in the tumour and/or stroma regions were selected for verification by IHC on additional samples. Among these, TFRC and PDLIM3 emerged as the two proteins of interest, supported by both evidence derived from spatial proteomics and tissue microarrays. These two proteins have previously been reported in various cancers, such as endometrial, breast, and ovarian cancers, but have not been extensively studied for CP treatment.^38^, ^39^, ^43^, ^44^, ^45^ TFRC is vital for intracellular iron transport and is linked to ferroptosis, a form of cell death associated with drug resistance in cancer.^46^, ^47^ The induction of ferroptosis may reverse this resistance, as high TFRC expression is observed in chemotherapy‐sensitive cells.^48^ This study supports the use of ferroptosis to enhance the efficacy of HGSC treatment and suggests that modulating TFRC levels may be an effective strategy against drug resistance in various cancers. PDLIM3 was one of the PDLIM family; it was involved in cytoskeletal regulation through actin filament organization. Notably, the PDLIM family comprises 10 members,^49^ several of which—including PDLIM2^50^ and PDLIM4—have been linked to ovarian cancer progression via mechanisms such as the STAT3 signalling pathway.^51^ Collectively, these insights position PDLIM3 as a potential crucial factor in the prognosis and therapy of ovarian cancer, and our study supports its role as a key protein associated with CP sensitivity in HGSC. But understanding the mechanistic involvement of TFRC and PDLIM3 in drug resistance would be a valuable insight into predicting chemotherapy efficacy and guiding therapeutic strategies for ovarian cancer patients.

Despite the state‐of‐the‐art technology being employed, there are several limitations in this study. In technology, excision size of FFPE samples, analysis throughput and spatial proteome profiling and so on result in much unsatisfactory. With the fast development of spatial analysis in proteomics, the technological obstacles should be solved in the near future. In sample sizes in either spatial proteomes or tissue microarrays, the conclusions derived from this study appear not to be fully supported by a large cohort. At the initial phase of this study, we have realized the technical limitations of spatial proteomics, especially in analysis throughput,^52^, ^53^ which causes a difficulty in large sample analysis. To gain unbiased data from limited samples, we closely collaborated with medical doctors in the hospital to set a strict criterion for selection of the HGSC patients with higher similarity, as to effectively avoid the individual errors in patients. In addition, we accepted a dense collection of micro‐spots in a sample (approximately 12 spots in a specific region per sample and 100 spots collected in continuous mode in the region with mixed tumour and stroma per sample), to achieve a statistical evaluation of spatial proteomics from limited samples. Even though the effort was paid, the drawbacks in this study were not completely diminished. First, a cohort study with large samples will provide a natural profile of proteomic distribution, which is a solid base to establish a spatial map of proteomics in human tissue. Our study with limited samples only reflects the partial proteomics profiles of several samples, because the technical considerations in proteomics could improve the data quality, but could not represent the natural behaviour in biological samples. Second, the data quality based on some technologies is highly sample‐size dependent; for instance, the quantification data obtained from IHC image analysis is a typical case. Due to the variability of IHC staining intensity and the flexibility of staining area estimation by software, the cumulative quantification of IHC images is a challenging technique, which needs a large sample to reach statistical conclusions. Therefore, this study is the first attempt to utilize a new proteomics technology on ovarian cancer tissue and has achieved positive results that spatial proteomics is workable in the HGSC FFPE slides; however, the correlative study described above does not come to a conclusion for new HGSC biomarkers on FFPE, especially for clinical diagnosis.

## 4 MATERIALS AND METHODS

### 4.1 Collection of FFPE samples from the HGSC patients

Ten patients diagnosed with stage III‐IV HGSC were recruited from the Department of Gynecologic Oncology, Zhejiang Cancer Hospital (Table 1; Supporting Information Data S1). They underwent primary debulking surgery, and residual tumour were all at R0 level in 2016–2019. Then, among the ten patients, nine of them underwent platinum‐based chemotherapy (carboplatin and paclitaxel) for 5–8 cycles, except for one patient who relapsed after two cycles of CP chemotherapy. Based on PFI, 10 patients were selected and divided into CP‐s and CP‐i. CP‐s were patients whose PFI is >24 months after chemotherapy, and CP‐i were patients who relapsed within 6 months after chemotherapy. All CP‐i patients died within 24 months, whereas patients in the CP‐s group were all alive before our last follow‐up. In particular, two patients (s2 and s5) did not experience recurrence until the last follow‐up. The last follow‐up period for CP‐s patients ranged from 2023/2/7 to 2023/6/6. Therefore, we calculated both the progression‐free survival (PFS) and overall survival (OS) until the last follow‐up. Furthermore, as shown in Table 1, the mean PFS of the CP‐i group was 6.35 months (min: 3.17, max: 9.87), and that of the CP‐s group was 52.53 months (min: 33.03, max: until the last follow‐up time). The mean OS of the CP‐i group was 13.40 months (min:9.03, max: 19.30), and that of the CP‐s group is 65.21 months (all alive). This study was conducted in accordance with recognized ethical guidelines, and the protocol for patient recruitment and sample collection was approved by the hospital's ethics committee in hospital (IRB2022410). Informed consent was obtained from all the patients.

**TABLE 1:** Survival information of 10 patients with HGSC that provided samples for spatial proteomics.

Patient ID	Age, years	Stage (FIGO)	Group	PFI, m	PFS, m	OS, m
i1	41	IVB	CP‐i	2.23	7.07	9.13
i2	75	IVB	CP‐i	.8	3.17	9.03
i3	60	IIIC	CP‐i	.90	5.73	18.13
i4	49	IVB	CP‐i	4.57	9.87	19.30
i5	40	IIIC	CP‐i	.67	5.90	11.43
Mean	53 ± 14.68	/	/	1.83 ± 1.64	6.35 ± 2.43	13.40 ± 4.96
s1	46	IIIC	CP‐s	56.83	61.47	84.87
s2	44	IVB	CP‐s	44.45	48.33	48.33
s3	55	IIIC	CP‐s	27.37	33.03	61.30
s4	73	IIIC	CP‐s	35.67	40.03	51.73
s5	75	IVB	CP‐s	74.33	79.80	79.80
Mean	58.6 ± 14.67	/	/	47.73 ± 18.45	52.53 ± 18.56	65.21 ± 16.44

Abbreviations: PFI, platinum‐free interval; PFS, progression free survival; OS: overall survival; CP‐s, carboplatin+paclitaxel‐sensitive, CP‐i, carboplatin+paclitaxel‐insensitive.

### 4.2 Spot excision and collection by LCM

The FFPE‐preserved tissues were sliced by FFPE microtome (Leica, Germany), with a thickness of 5 µm. The excised slices were salvaged onto PET membrane (Leica) and incubated at RT overnight. The FFPE slices were dewaxed in 100% xylol for 5 min and were hydrated in an ethanol gradient of 100%, 95%, to 70%. Dewaxed slices were incubated with hematoxylin for 3 min and were differentiated with 1% hydrochloric acid in ethanol for 30 s, followed by a thorough rinse with water. The treated slices were covered by a thin layer of 1% eosin for 30 s. After HE staining, a pathologist carefully checked the stained slides and labelled typical regions such as tumour, stroma, and mixed regions with tumour and stroma. For the specific regions of tumour or stroma, a pathologist carefully checked the quality of HE images for all the FFPE slides from the 10 patients, and tried to define the regions with typical images of tumour or stroma characteristics (in fact, such regions were not found in all the samples). Those spots in the specific regions were individually excised at 0.1 mm^2^/spot. For the common regions mixed with tumour and stroma, all the spots within the region were cut by LCM in continuous mode with 0.1 mm^2^/spot at 10 spots per line. The microdissection was implemented in the Arcturus Collect (Applied Biosystems) with parameters set at UV laser power 80% and UV cutting speed 51. The excised spots were transferred onto the caps of 200 µL Eppendorf tubes for further treatment next step.

### 4.3 Extraction and digestion of proteins on FFPE spots

The micro‐dissected spots were subjected to protein extraction and digestion within the caps of Eppendorf tubes using a previously described protocol from our laboratory.^28^ Briefly, after 30 µL of 25 mM ammonia bicarbonate (NH_4_CO_3_) was added to the cap, the spots were incubated in a heating block at 95°C for 1.5 h for de‐crosslinking, and were placed into a water bath at 37°C overnight with addition of 1 µL of 50 ng/µL trypsin without prior treatment of alkylation and reduction. To terminate digestion, the spots were incubated with 1 µL of 10% FA, followed by centrifuging of dryer to reduce the volume to 10–15 µL.

### 4.4 Separation and identification of peptides using LC‐MS/MS

The tryptic peptides were loaded on a PepMap Neo C18 trap column (300 µm × 5 mm, particle size of 5 µm, 100 Å; ThermoFisher) and were separated using a NanoElute system (Bruker Daltonics) wherein a house‐made C18 column (75 µm × 25 cm, particle size of 1.9 µm, 100 Å) was mounted. Under column temperature at 50°C, peptides were eluted using a linear 30 min gradient, 2%–22% buffer B (buffer A: 0.1% FA and 99.9% ddH_2_O, and buffer B: 0.1% formic acid and 99.9% acetonitrile) within 0–20 min, 22%–37% buffer B in 20–24 min, 37%–80% buffer B in 24–26 min, and 80% buffer B in 26–30 min. The eluted peptides were delivered to timsTOF Pro2 (Bruker Daltonics), and the spectra corresponding to the peptides were acquired in DIA mode. Based on the dia‐PASEF method, an MS1 scan was further treated with 12 dia‐PASEF scans with two ion mobility windows per dia‐PASEF scan, covering an m/z range of 452–1152 and ion mobility of.76 to 1.29 Vs cm^−2^.

To evaluate the data quality of LC‐MS/MS, *E. coli* lysate as a standard control was measured daily, and the peptides identified and their retention times were monitored and examined daily. To evaluate the data quality in entire proteomes, the correlation coefficient of pairwise Spearman's among all quality‐control runs was assessed using R (v.3.2.129) (Figure S1A).

### 4.5 Peptide annotation based on MS/MS signals

The raw data acquired by the timsTOF Pro2 MS were processed using Spectronaut (version 16.1.220730) in DirectDIA mode according to the peptides annotated from the UniProt database (2022 release, UP000005640_9606). For an identified peptide upon MS/MS, the minimal length was set at 7 amino acids and missed cleavages≤2. A MS/MS spectrum matched to a peptide or protein was judged by FDR ≤1%. Protein quantification of DIA signals was performed using the MaxLFQ algorithm in the R package iq v.1.9.6. Protein quantification was globally normalized to all the samples using the R package limma (v.3.58.1), during which the non‐zero minimum value was filled in the protein expression matrix.

### 4.6 Tissue microarrays with immunohistochemistry

A tissue microarray panel was created at the Zhejiang Cancer Hospital (Supporting Information Data S1). Antibodies against IHC were purchased from two sources: ALDH5A1 (ab129017), TFRC (ab214039), PDLIM3 (ab224335 and CA1 (ab267475) from Abcam, and APM2 (YN4241) from Immunoway. The IHC staining kit was gained from MXB (Fuzhou Maixin Biotech Co.), including MaxVivsionTM 3 HRP kit (KIT‐5220); all the IHC processes were followed with the manufacturer's instructions. The IHC staining images were obtained using InstantViewer (3DHISTECH), and the image analysis primarily relied on the Multiplex IHC v3.4.9 algorithm in HALO software (Indica Labs), which quantified cells positive for tumour or stroma regions recognized in the images based on staining intensity.

### 4.7 Bioinformatics analysis

#### 4.7.1 Quality assessment for the spatial proteomics data

Using geom_density in ggplot2 (v.3.5.0), a probability curve of protein intensities in a spot was generated based on the abundance of identified proteins, and the distribution of all spots in a sample was appraised to define the main trend of the probability curves in total spots. A spot with an obvious shift from the main trend was considered abnormal and was removed.

#### 4.7.2 Analysis of individual differences among FFPE samples

Two approaches were implemented to reduce the influence of individual differences in spatial proteomes between samples. First, random sampling statistical analysis was performed to evaluate the individual differences in proteomes in the tumour and stroma in all the samples. A set of proteins was randomly picked from two samples in the same group (tumour or stroma), and the abundance differences of these proteins between the two samples were estimated by the Wilcoxon test. A protein in two samples with *p*‐value <.05 and log2FC > 1 was defined as a DEP. After over 1000 random samplings, a DEP was identified more than 10 times and was assigned as an individual DEP. All non‐insignificantly different proteins in a group (tumour or stroma) were pooled for subsequent analysis. Second, harmony analysis was performed to reduce the individual differences in the proteomes in continuous regions. Briefly, the proteins in all the spots with an abundance mean > 2.5 and standard deviation > 1 were selected for PCA, and the PCs with a cumulative variance percentage over 40% were subjected to the R package harmony (v.1.2.0).^54^ The transformed PCs matrix after the harmony treatment is sent to the R package ConsensusClusterPlus (v.1.66.0) for further clustering. With 5000 iterations, a stable K‐means clustering result was achieved, in which the transformed PCs were independent of individual differences. To explore the proteins involved in these clusters, random forest and feature recursive elimination were implemented under the supervision mode using the R packages randomForest (v.4.7‐1.1) and caret (v.6.0‐94).

#### 4.7.3 Deconvolution for cell classification based on spatial proteome

CIBERSORTx (https://cibersortx.stanford.edu/) was used for the deconvolution analysis. During the deconvolution, a matrix was built, including the spatial proteomics of each spot or region and cell features upon transcriptome analysis.^55^ To identify cell‐dependent proteins, a database was constructed in our laboratory, which contained a set of genes whose transcriptome and proteome possessed a well‐accepted abundance correlation in single‐cell type based on public databases and literature. Subsequently, a new spatial proteomics dataset was created by overlapping spatial proteomics per spot or region, and abundance‐correlated genes between transcripts and proteins. According to Qian et al.,^13^ regarding scRNA‐seq on ovarian cancer, the cells in the tumour tissues were broadly clustered into six cell types based on the different transcriptome abundance among these clusters as cell‐dependent transcript features. Finally, a new spatial proteomics data set and cell‐dependent transcript features were input into CIBERSORTx for deconvolution analysis.^55^

#### 4.7.4 ssGSEA of spatial proteomics

ssGSEA was performed using the R package GSVA (v.1.50.2). The DEPs between the tumour and stroma or drug‐sensitive or drug‐insensitive regions in all the samples were input into the program, and the overall adjusted DEP ranks were estimated. ssGSEA values guided the generation of tumour scores and chemical scores.

#### 4.7.5 Functional annotations of spatial proteomics

All the biologically functional annotations of the spatial proteomics in this study were performed with the R package clusterProfiler (v.4.10.1), focusing on GO‐BP.^56^ To trace the trends in protein abundance changes among different clusters, Mfuzz (v.2.62.0) was employed with fuzzy c‐means to determine the abundance change patterns.

#### 4.7.6 Hierarchical analysis of spatial proteomics

PCA and t‐distributed stochastic neighbour embedding were used to evaluate the proteome differences of samples or groups through dimensional reduction and comparison, and visualization using the R package stats (v. 4.3.1) and tsne (v. 0.17). K‐means and heatmap clustering were employed to overall appraise the similarities or differences among spots or samples based on spatial proteomics data using the R packages of ConsensusClusterPlus (v. 1.66.0) and ComplexHeatmap (v. 2.18.0).

#### 4.7.7 Survival analysis

The survival analysis was based on the Kaplan–Meier database (https://kmplot.com) that contains HGSC samples with the corresponding mRNAs and clinical information.

#### 4.7.8 Statistics

Considering the data distribution patterns, the Wilcoxon test and Student's *t*‐test were used to assess the significant differences among samples, using the R package stats (v. 4.3.1).

## AUTHOR CONTRIBUTIONS

Linyuan Fan: Conceptualization, methodology, writing—original draft. Liu Yi: Resources, writing—original, draft. Haichao Zhou: Visualization, writing—original draft. Yang Feng: Methodology, data curation. Guangyi Jiang: Resources. Guixue Hou: Investigation. Zhihan Cao: Formal analysis. Zhiguo Zheng: Resources. Lu Sun: Resources. Hao Chen: Methodology. Yuefei Zhang: Methodology. Weiran Chen: Resources. Yun Xi: Formal analysis. Benliang Cheng: Validation. Qinghai Yang: Validation. Yan Ren: Writing—review & editing, supervision. JianQing Zhu: Resources, supervision, project administration, funding acquisition. Siqi Liu: Writing—review & editing, supervision, project administration, funding acquisition. All authors approved the final version of the manuscript.

## CONFLICT OF INTEREST STATEMENT

The authors declare no conflict of interest.

## ETHICS APPROVAL AND CONSENT TO PARTICIPATE

This study was approved by the Medical Ethics Committee of the Zhejiang Cancer Hospital (IRB2022410). The methodology employed in this study adhered to the ethical standards outlined in the Declaration of Helsinki. Informed consent was signed before sample collection.

## Supporting information



Supporting Information

Supporting Information

Supporting Information

Supporting Information

Supporting Information

Supporting Information

Supporting Information

Supporting Information

Supporting Information

Supporting Information

Supporting Information

Supporting Information

## Data Availability

The raw proteomics data generated during this study were archived in the iProX database and are accessible via the following identifier: PXD060611, also available as IPX0010963000.
