# Supplementary figures and images for: Spatially resolved proteomics surveys the chemo‐refractory proteins related to high‐grade serous ovarian cancer
Source: Clin Transl Med. 2025 Jul 23;15(7):e70422. doi: 10.1002/ctm2.70422 (PMC12284440; doi:10.1002/ctm2.70422)

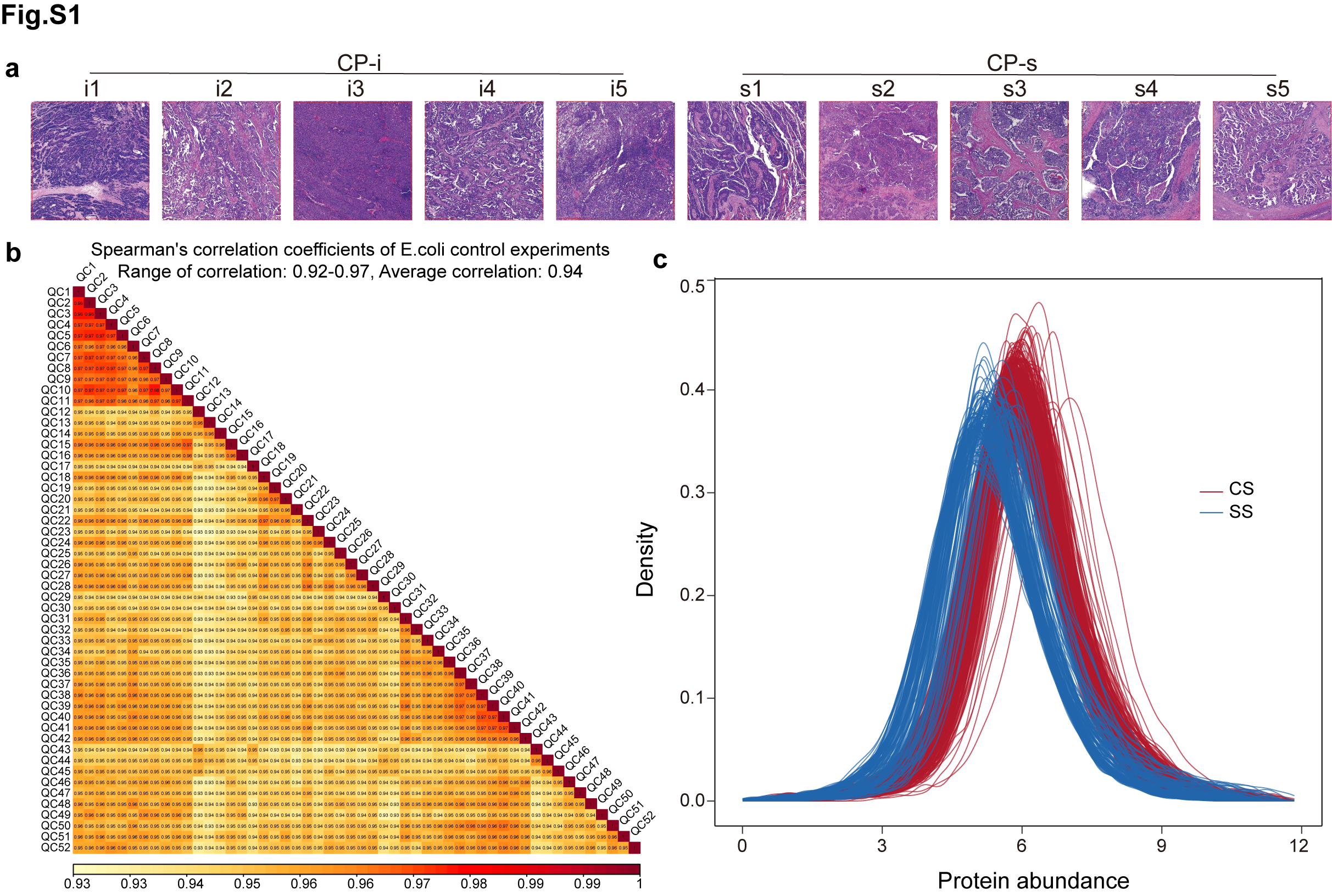

Supplement: Supplementary file 1 — Supporting Information [file CTM2-15-e70422-s002.tif]

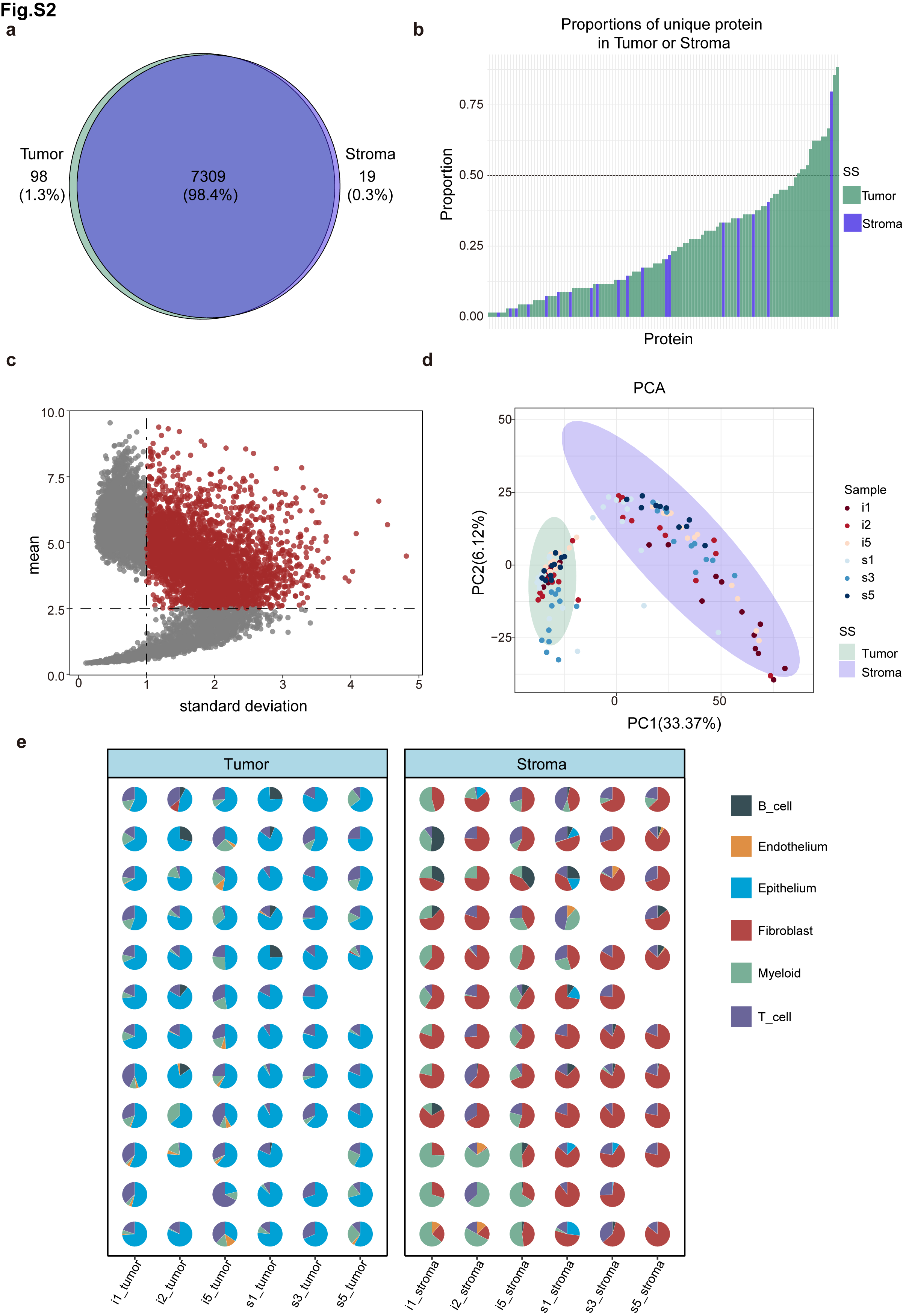

Supplement: Supplementary file 2 — Supporting Information [file CTM2-15-e70422-s009.tif]

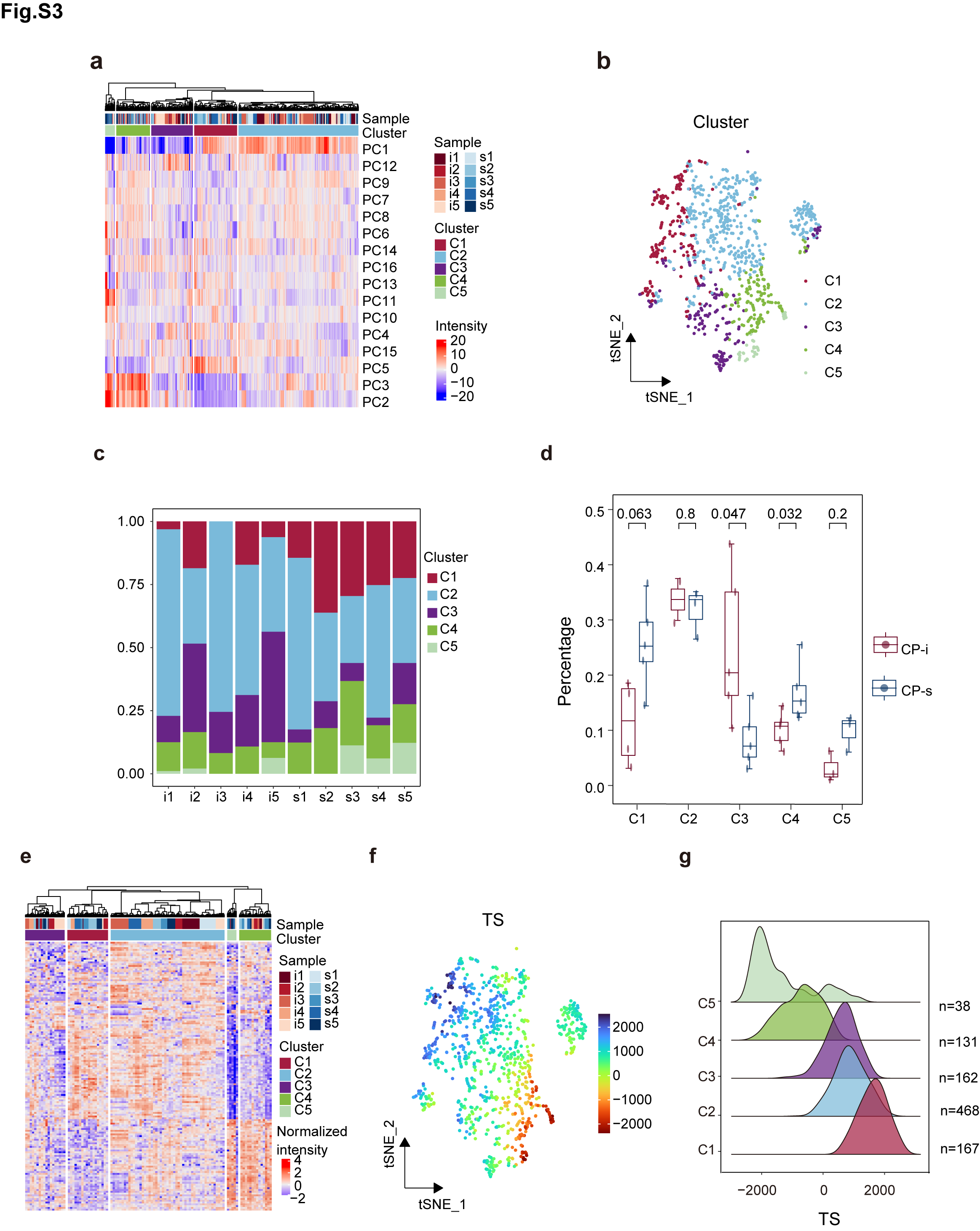

Supplement: Supplementary file 3 — Supporting Information [file CTM2-15-e70422-s011.tif]

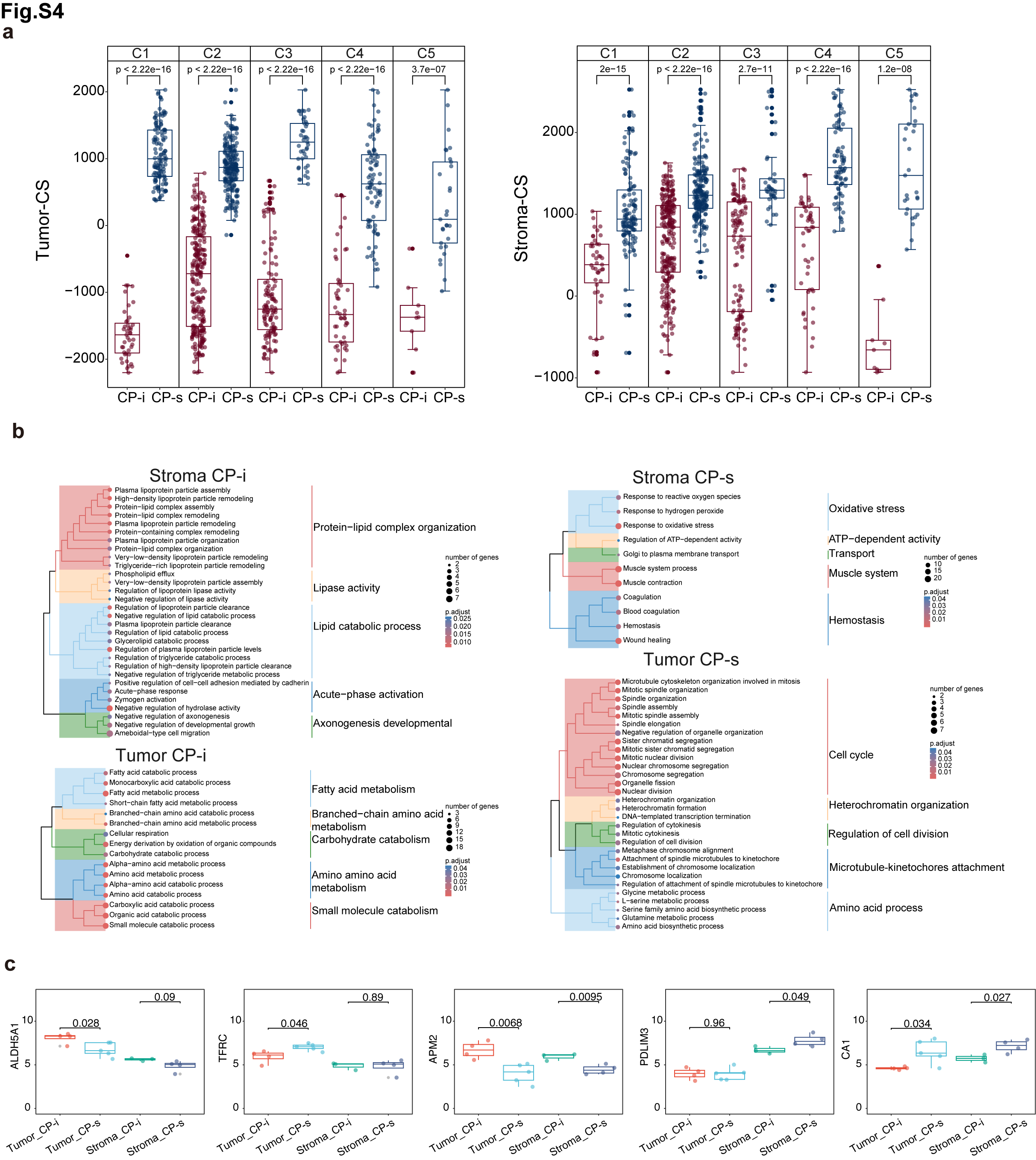

Supplement: Supplementary file 4 — Supporting Information [file CTM2-15-e70422-s005.tif]

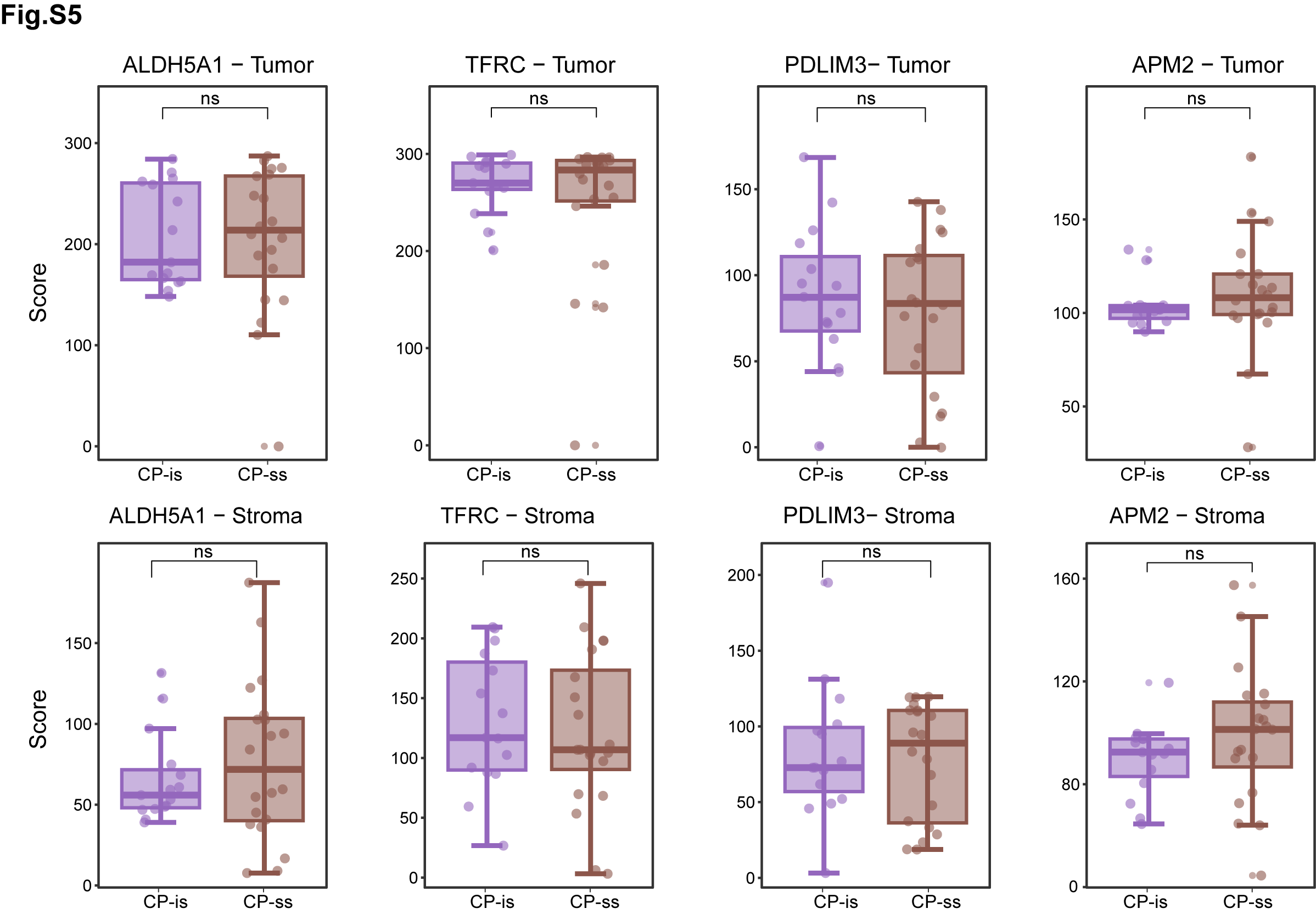

Supplement: Supplementary file 5 — Supporting Information [file CTM2-15-e70422-s004.tif]

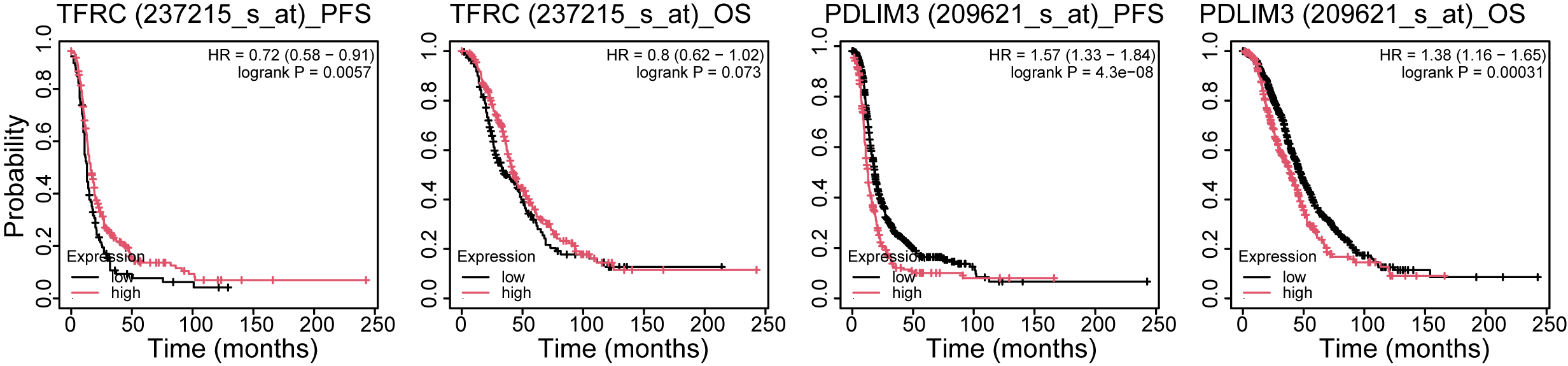

Supplement: Supplementary file 6 — Supporting Information [file CTM2-15-e70422-s001.tif]
