# Supplementary material for: Spatially resolved proteomics surveys the chemo‐refractory proteins related to high‐grade serous ovarian cancer
Source: Clin Transl Med. 2025 Jul 23;15(7):e70422. doi: 10.1002/ctm2.70422 (PMC12284440; doi:10.1002/ctm2.70422)
Supplement: Supplementary file 12 — Supporting Information [file CTM2-15-e70422-s006.docx]

Supplementary Information

**Spatially resolved proteomics surveys the chemo-refractory proteins related to high-grade serous ovarian cancer**

*Linyuan Fan^1,2,3,7^, Yi Liu^4,7^, Haichao Zhou^1,2,3,7^, Yang Feng^1,2,3^, Guangyi Jiang^4^, Guixue Hou^2^, Zhihan Cao^1,2,3^, Zhiguo Zheng^4^, Lu Sun^4^, Hao Chen^2^, Yuefei Zhang^1,2,3^, Weiran Chen^3^, Yun Xi^4^, Benliang Cheng^6^,* *Qinghai Yang^6^**, Yan Ren^3,5,*^, Jianqing Zhu^4, *^,Siqi Liu^1,2,3,*^*


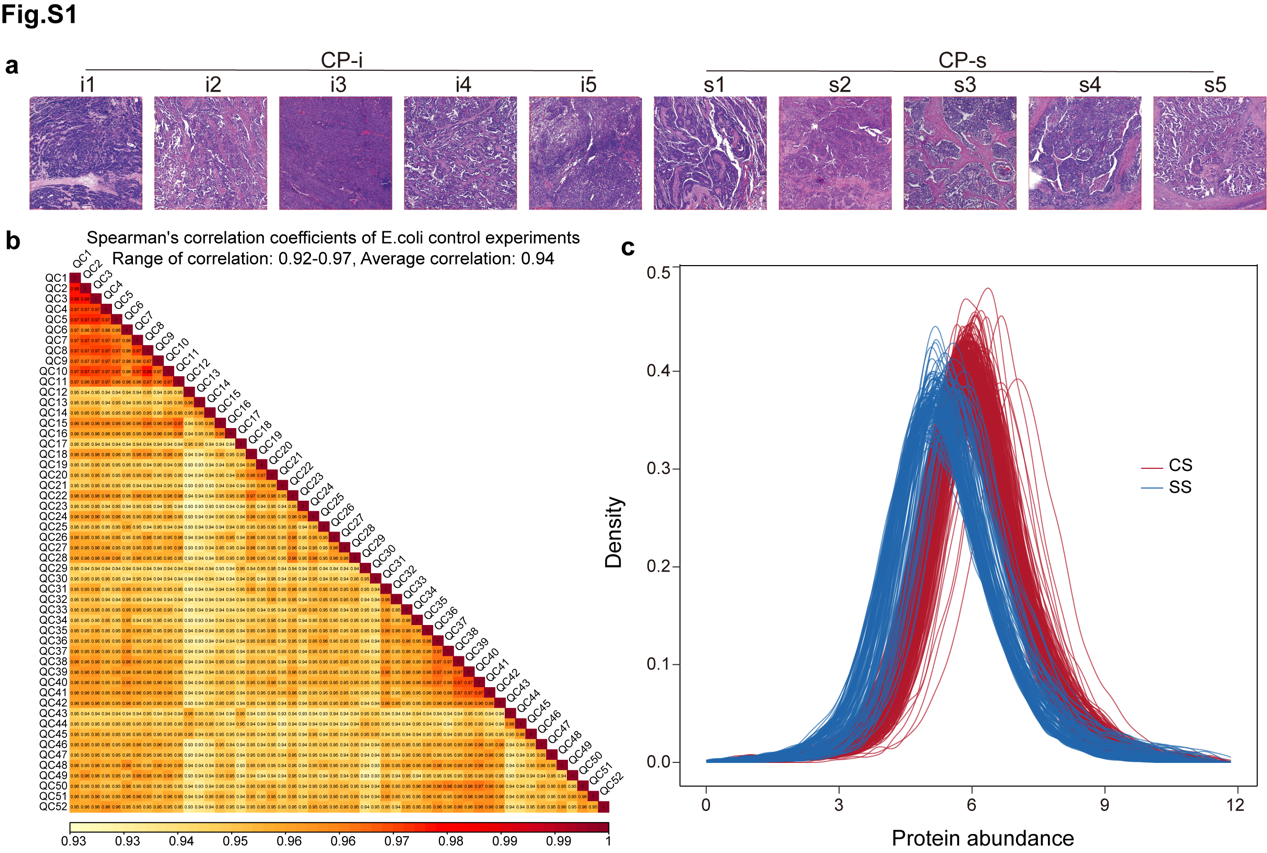


Fig.S1. Data quality control. a) The H&E-stained image from 10 patients of the continuous sampling containing 100 spots. b) Correlation matrix of 52 E coli proteomes (Spearman’s correlation coefficients). The color gradient from yellow to red indicating correlation values ranging 0.93 to 1. Spearman's correlation coefficients of E.coli control experiments ( range of correlation: 0.92-0.97, average correlation: 0.94). c) Distribution of protein abundances in CS (red) and SS (blue) by a density plot. A unimodal distribution (dip test) was observed.


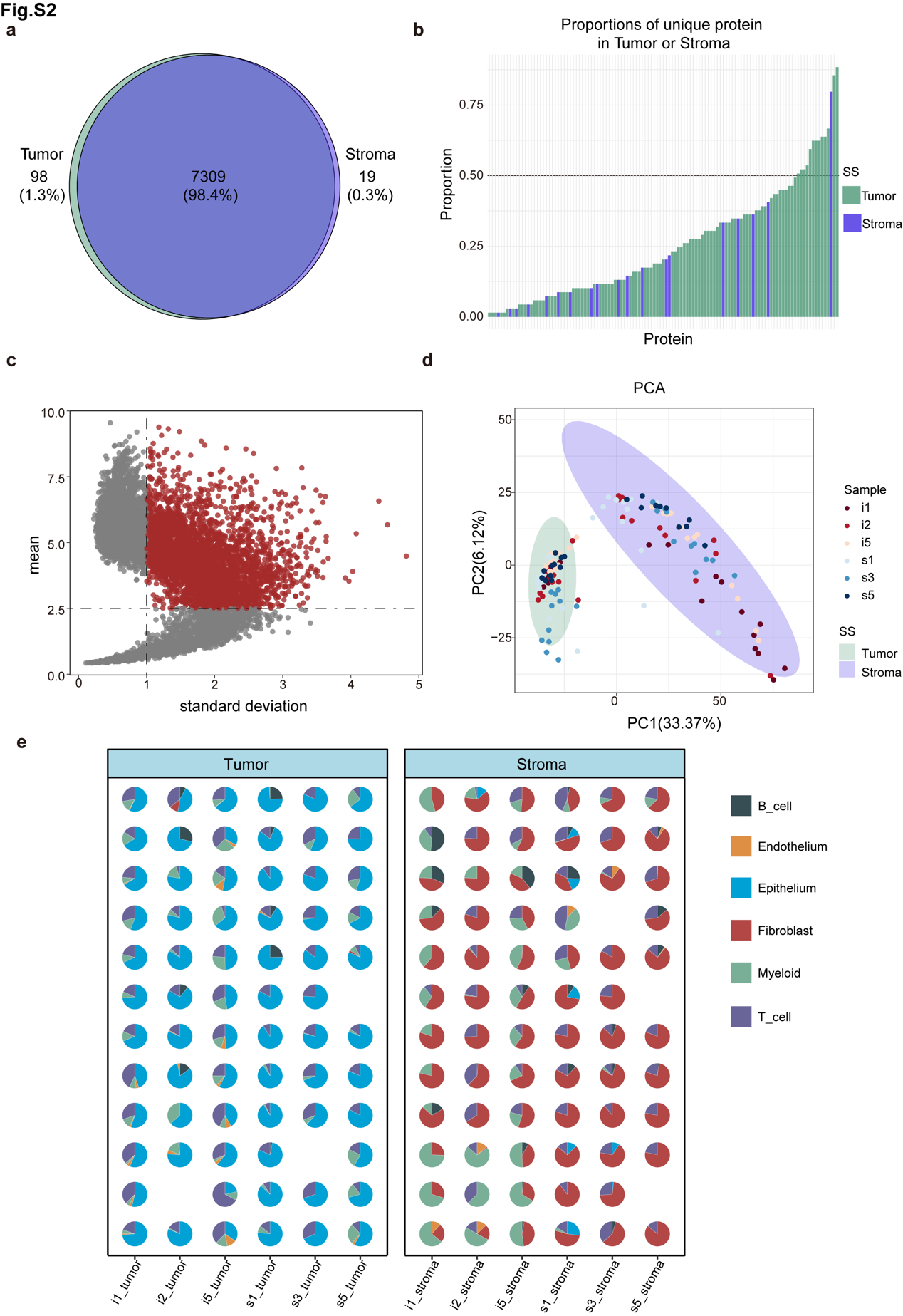


Fig.S2. a) Overlap of proteome identification between tumor and stroma, tumor in green, and stroma in purple. b) Proportion of unique proteins in spots of tumor or stroma region, tumor in green, and stroma in purple. c) Proteins with stable expression of all spots in specific regions. The horizontal axis is standard deviation (sd) of each protein in all samples, the vertical axis is Mean of each protein in all samples, the threshold value of mean of gray horizontal line is 2.5, the threshold value of sd of gray vertical line is 1, and the red part is preserved protein. d) PCA of specific region data. Horizontal and vertical axis are first and second principal component of PCA, respectively. Dots represent tissue samples. e) Cell deconvolution for every spot either tumor or stroma in a sample with spatial proteomics. A pie chart stands for the ratios of cell types labeled by different colors in a spot.


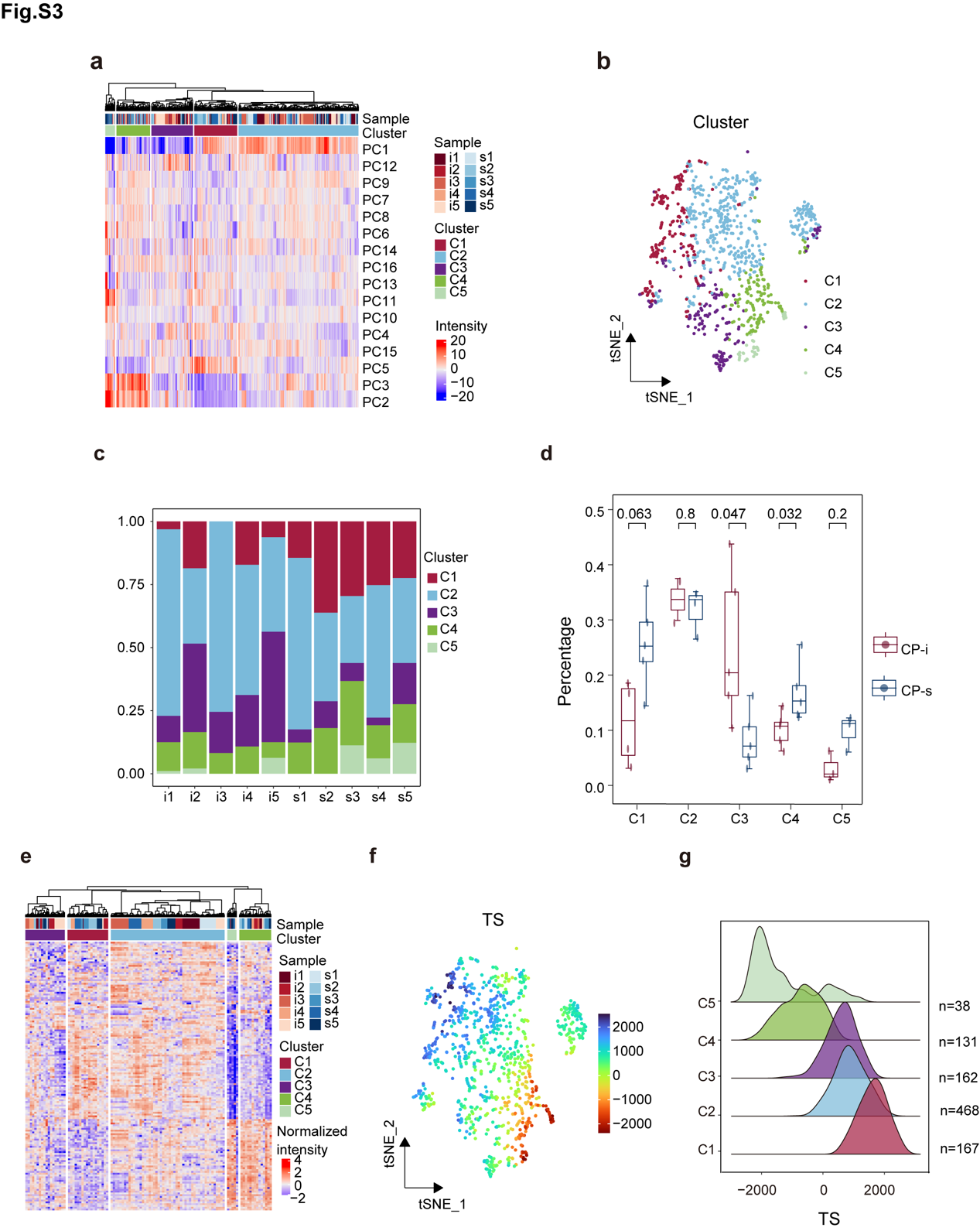


Fig.S3 a) Heatmap of the continuous region data based on principal components (PCs). The horizontal axis represents different samples, with the upper color indicating sample classification: green for tumor region samples and purple for stroma region samples. The vertical axis represents PCs derived from PCA analysis. Each spot on each PC has a corresponding coordinate value, which represents the value in the heatmap. b) tSNE of continuous region data after Harmony treatment, colored by clusters. c) Statistics on the ratio of clusters. Colors represent different clusters, with the horizontal axis denoting the ratio and the vertical axis showing samples. d) Comparison of cluster ratios between CP-i and CP-s, with values on the subgraphs representing p-values from the Wilcox test. The horizontal axis represents ratios of each cluster in CP-i or CP-s group, the vertical axis represents two groups in different clusters, and colors indicate different groups. Ratio of each cluster for each group are represented as boxes. Each box represents the quartiles of the data, with the line inside the box indicating the median. The upper and lower edges of the box represent the first and third quartiles, respectively. e) Distribution of 450 proteins across 5 clusters in spots from the continuous region. The horizontal axis represents different samples, with the upper color indicating sample classification and the lower color indicating clusters. The vertical axis represents proteins arranged according to hierarchical clustering results. Quantitative data are normalized, with colors from blue to red indicating values from low to high. f) tSNE of continuous region data after Harmony treatment, colored by TS. The color gradient from red to blue represents the TS values of spots ranging from -2000 to 2000. g) Density plot of TS for spots in each cluster. The horizontal axis represents TS, the vertical axis denotes clusters, and colors indicate different clusters. TS values corresponding to spots within the same cluster form a curve.


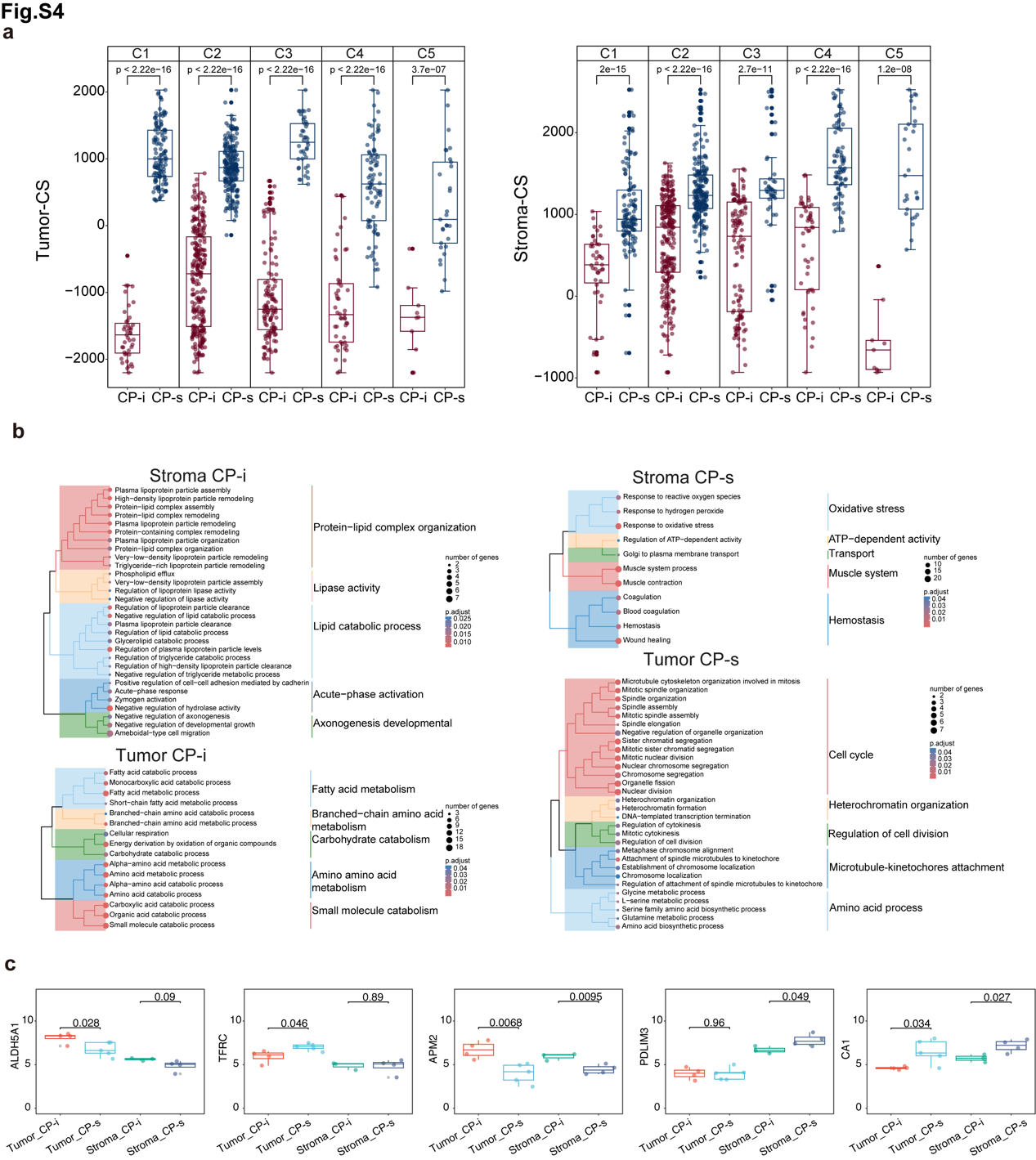


Fig.S4. a) Comparison of cluster-related CS between CP-i and CP-s. The horizontal axis represents CS values, the vertical axis represents the group, and colors represents different groups. CS values of spots within each cluster for each group are represented as boxes. Each box represents the quartiles of the data, with the line inside the box indicating the median. The upper and lower edges of the box represent the first and third quartiles, respectively. b) The biological process annotations for the upregulated DEPs from CP-i and CP-s of tumor and stroma, respectively. Each line represents a single annotated pathway, with different pathways with similar functions displayed in the same color on the left. The summarized functions correspond to the color line on the right. The size of the circles indicates the number of genes, while the gradient from red to blue reflects the adjusted *p*-values, ranging from small to large. c) Paired comparison for the protein abundance of 5 targets between CP-i and CP-s groups in the tumor or stroma based on Student’s t-test.


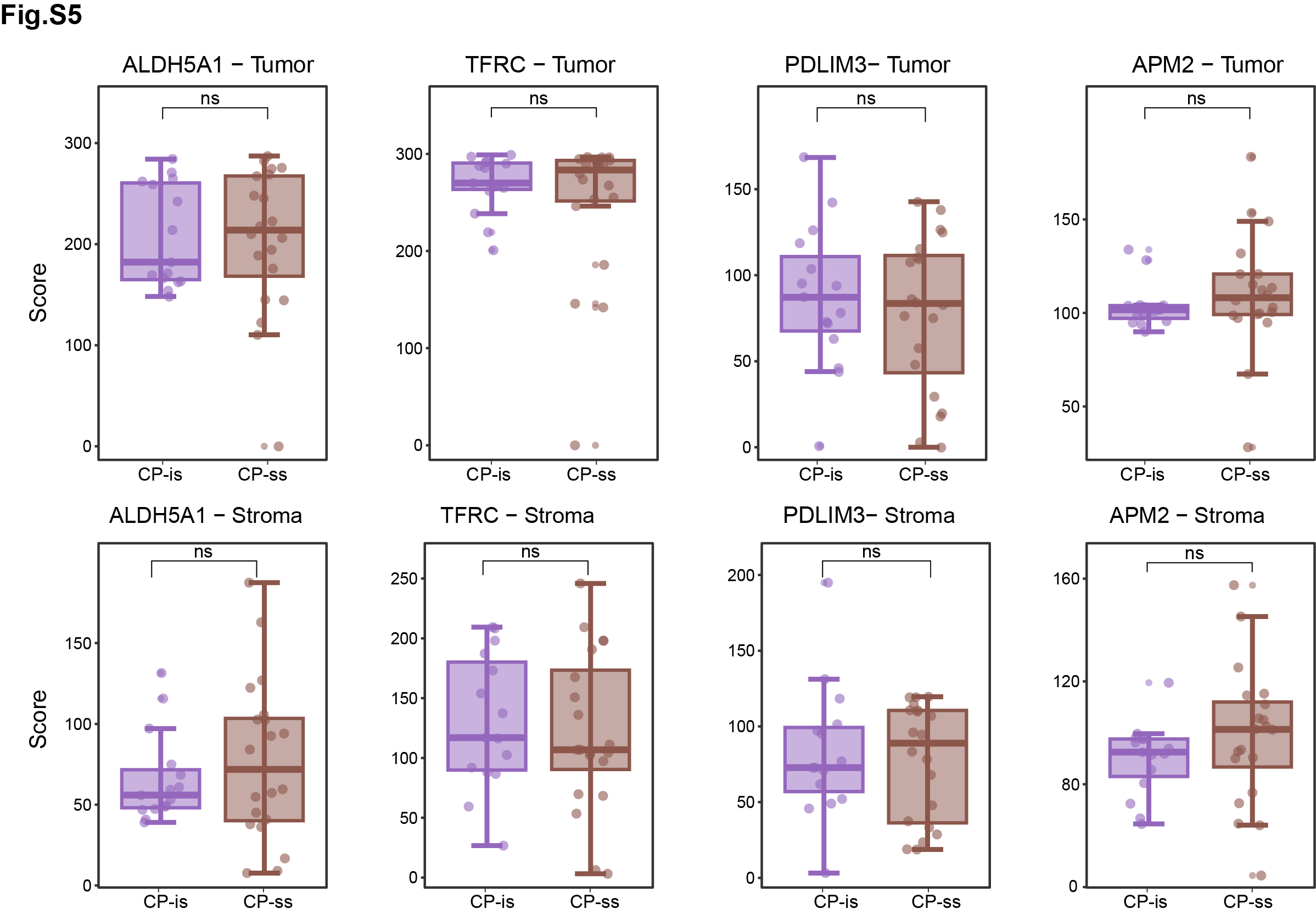


Fig.S5. Paired comparison for the IHC staining intensities of 4 targets between CP-is and CP-ss in all the samples of the tissue microarrays based on Student’s t-test. On upper and lower panel, comparison of 4 targets between CP-ip and CP-sp from tumor and stroma, respectively. The signs of *, **, ***, and ns represent a significance with *P*<0.05, *P*<0.01, *P*<0.001, and non-significance.


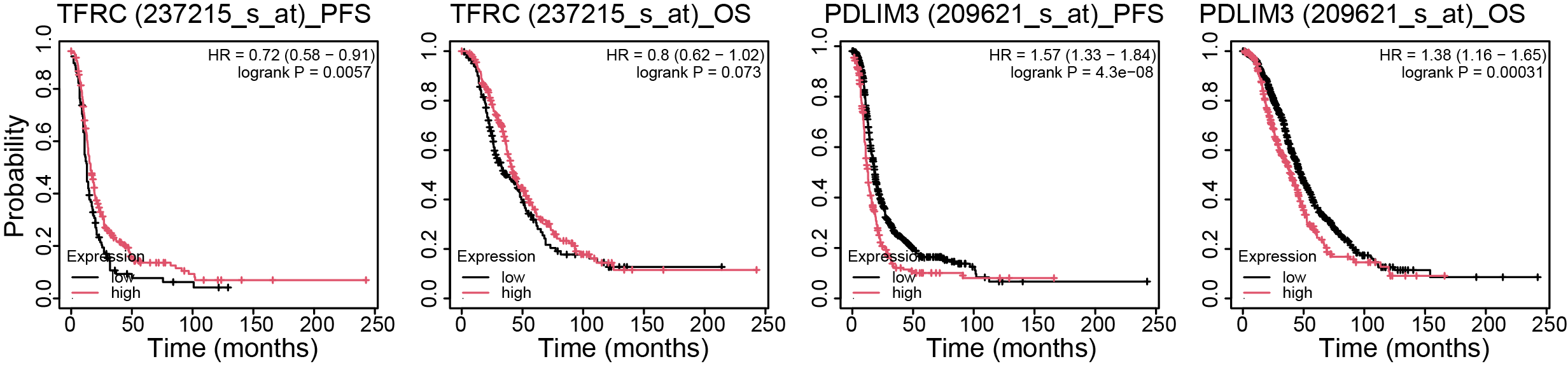


Fig.S6. PFS and OS analysis of TFRC and PDLIM3 based on RNA array. The vertical ordinate represents survival time, and the horizontal ordinate represents survival probability.
